# Phenolic-rich *Rubus caesius* roots from Turkmenistan: *in vitro* antioxidant, enzyme inhibition, antibacterial, and antifungal properties and *in silico* analysis

**DOI:** 10.1039/d6ra04860a

**Published:** 2026-08-11

**Authors:** Serdar Korpayev, Emre Can Buluz, Savas Kaya, Jasmina Glamočlija, Neda Popović, Uroš Gašić, Dejan Stojković, Hemra Hamrayev, Mirap Agamuradov, Sakina Yagi, Gokhan Zengin

**Affiliations:** a Biotechnology Institute, Ankara University 06100 Ankara Turkey; b Institute of Science, Department of Physics, Ege University İzmir 35100 Turkey; c Sivas Cumhuriyet University, Faculty of Science, Department of Chemistry 58140 Sivas Turkey savaskaya@cumhuriyet.edu.tr; d Department of Plant Physiology, Institute for Biological Research “Siniša Stanković” – National Institute of Republic of Serbia, University of Belgrade Bulevar Despota Stefana 142 11108 Belgrade Serbia; e Department of Chemical and Petroleum Engineering, University of Calgary 2500, University Drive NW Calgary Alberta T2N 1N4 Canada; f Saint Petersburg State Pediatric Medical University of the Ministry of Health of the Russian Federation Russian Federation; g The Department of Virology and Microbiology, Turkmen State Medical University Named After Myrat Garryev Ashgabat Turkmenistan; h Department of Biology, Science Faculty, Khartoum University Khartoum Sudan; i Department of Biology, Science Faculty, Selcuk University Konya Turkey

## Abstract

The dewberry, or *Rubus caesius* L. (Rosaceae), has long been used in traditional medicine, although scientific research has primarily focused on its roots and aerial portions. Notable biological activity associated with a rich phytochemical profile has been reported in these investigations. In contrast, limited information is available regarding the biological potential and chemical composition of *R. caesius* roots collected from Turkmenistan, Central Asia. The present study aimed to investigate, for the first time, the biological potential of *R. caesius* root extracts prepared as an aqueous infusion (a traditional, food-relevant preparation) and an ethanol extract (a polyphenol-enriching extraction), and to relate differences in bioactivity to UHPLC-QqQ-MS/MS-based phytochemical composition. The extracts were screened using complementary antioxidant assays (radical-scavenging and reducing power), a multi-target panel of enzyme inhibitory activities relevant to metabolic and cosmetic/neurobiological pathways (including α-amylase, α-glucosidase, acetyl-/butyrylcholinesterase, and tyrosinase), and antimicrobial/antifungal tests against representative bacterial and fungal strains. UHPLC-QqQ-MS/MS profiling revealed a root metabolome dominated by phenolic acids, flavonoids, and anthocyanin-related constituents, with clear qualitative and quantitative differences between infusion and ethanol extracts. In agreement with the chemical findings, both extracts expressed pronounced antioxidant capacity, while enzyme inhibition was extract-dependent, indicating that solvent polarity influenced the recovery of constituents responsible for carbohydrate-hydrolyzing enzyme and cholinesterase/tyrosinase inhibition. The antimicrobial and antifungal screenings demonstrated selective growth inhibition that varied by microorganism and extraction type. The integrated chemical–biological approach highlights *R. caesius* roots from Turkmenistan as a promising source of multifunctional natural products and provides baseline data to support their future use in nutraceutical, functional food, or phytopharmaceutical development.

## Abstract 2

*Rubus caesius* roots can be considered effective sources of bioactive agents in preparing functional applications. Image created with the assistance of Google Gemini.

## Graphical abstract

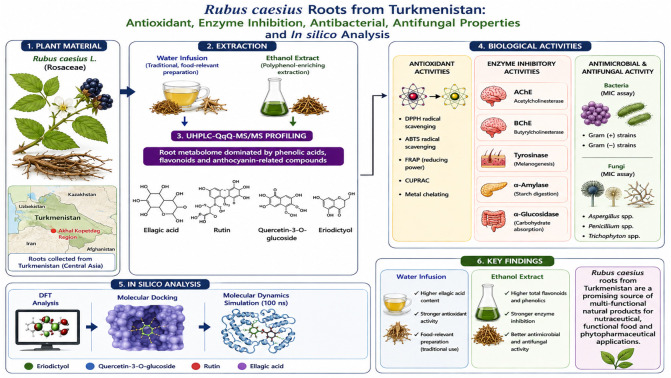

## 1 Introduction

*Rubus* species (Rosaceae) have historically been esteemed for their culinary and therapeutic applications, and contemporary phytochemical research has substantiated that numerous members of this genus are abundant in phenolic acids, flavonoids, anthocyanins, and hydrolyzable tannins, particularly ellagitannins, which can enhance a wide array of bioactivities.^1^*Rubus caesius* L. (European dewberry) is a widely distributed bramble in Europe and parts of Asia, such as Turkmenistan; however, it has been studied less actively than commercial *Rubus* crops, despite its availability as a wild resource.^1–3^ Traditionally, *Rubus* roots are ingested either fresh or processed, while preparations of the leaves and stems, including simple water infusions, are frequently used in folk practices, thereby justifying the exploration of “tea-like” extracts that mirror actual consumption practices.^4,5^

Oxidative stress and associated inflammatory pathways are linked to the advancement of several chronic diseases, while dietary polyphenols are often regarded as protective phytochemicals due to their ability to scavenge radicals and their extensive redox-modulating properties.^6^ Concurrently, enzyme inhibition is a recognized pharmacological approach to regulate essential biochemical pathways pertinent to common ailments: the inhibition of α-amylase/α-glucosidase is associated with postprandial glycemic regulation, acetylcholinesterase/butyrylcholinesterase inhibition pertains to cholinergic modulation in neurodegenerative diseases, and tyrosinase inhibition is extensively employed as a screening target for melanogenesis-related purposes.^4,7^ Evidence suggests that *R. caesius* extracts and fractions exhibit significant inhibitory effects across various enzyme panels, as well as antioxidant properties, indicating that this species may offer a multi-target phytochemical profile deserving of further investigation.^4^ Simultaneously, antimicrobial resistance (AMR) is recognized as a significant threat to global public health, making it increasingly urgent to investigate novel chemical scaffolds and multi-component natural products that may act through multiple pathways or complement current treatments.^8^ The WHO's list of fungal priority diseases, which attempts to direct research and innovation toward high-impact fungal concerns, reflects the growing significance of fungal infections and antifungal resistance. Continued screening of phenolic-rich taxa such as *Rubus* is supported by numerous reviews of plant phenolics (including flavonoids and tannins) as potential antimicrobial/antifungal agents with mechanisms that may include membrane disruption, enzyme inhibition, interference with quorum sensing/biofilms, and oxidative imbalance in microbial cells.^9,10^

Chemically, *Rubus* matrices may include a variety of polymeric structures and phenolic subclasses, which makes direct attribution of activity more difficult unless bioassays are supported by robust analytical profiling.^11^ The importance of high-resolution chromatographic processes for this species is further supported by recent targeted and untargeted studies on *R. caesius* in particular, which have revealed complex secondary metabolite patterns, including flavonoid glycosides and phenolic/tannin-related components.^12^ Notably, it has been demonstrated that the quantitative polyphenolic profiles of water and ethanol extracts from young leaves and stems differ (*e.g.*, enrichment of specific flavonoid glycosides/aglycones in ethanol), and these discrepancies can lead to distinct biological response patterns.^1^ Furthermore, recent reports have shown that *R. caesius* leaf/stem extracts can inhibit clinically relevant bacteria and fungi, including MRSA-associated phenotypes and biofilm-related endpoints. This suggests that non-fruit organs may be pharmacologically relevant rather than just agricultural by-products.^1,13^

Despite these developments, research on *Rubus caesius* remains largely limited to its aerial parts. While the roots of other *Rubus* species have been found to possess large concentrations of phenolic compounds and tannins with notable antioxidant, antibacterial, and enzyme inhibitory effects, the roots of *R. caesius* have received very little research. A major gap in the literature is the lack of comprehensive chemical and biological information on *R. caesius* roots. Against this background, the present study provides the first integrated report on *R. caesius* roots from Turkmenistan, comparing an infusion (aqueous) and an ethanol extract through a commonly used solvent system that can recover a wider polarity range of phenolics and enhance extraction of less polar constituents^1^ (i) a battery of antioxidant assays, (ii) enzyme inhibitory testing relevant to metabolic, neurobiological, and cosmetic pathways, (iii) antibacterial and antifungal screening, and (iv) LC-MS-based chemical characterization to annotate major constituents and evaluate extraction-dependent fingerprints. Additionally, *in silico* analyses were conducted to investigate potential interactions between the specified biological targets and the discovered chemicals. This study highlights *R. caesius* roots as an understudied source of pharmacologically relevant natural chemicals and offers fresh insights into their bioactive potential by combining chemical profiling, biological testing, and computational techniques.

## 2 Materials and methods

### 2.1 Plant material and extract preparation

*Rubus caesius* roots were collected from Germab village in the foothills of the Akhal Kopetdag region, Turkmenistan (38°00′29.3″ N, 57°43′59.2″ E). Botanical identification was performed by Batyr Danatarov. Careful excavation was used to obtain root samples, which were then cleaned to get rid of any remaining soil and further examined. After collection, the cleaned roots were dried under suitable condition, and ground into a fine powder prior to extraction.

#### 2.1.1 Preparation of *Rubus caesius* root extracts

The extraction process involved two distinct methods. First, the infusion extract was created by mixing 10 g of finely ground *R. caesius* roots with 200 mL of boiling distilled water for 15 minutes, followed by filtration and freeze-drying. Second, the ethanolic extract was produced by stirring 15 g of the powdered root in 70% ethanol at room temperature. This extraction continued until the solvent's color faded, adhering to established protocols. The final stage involved filtering the suspension and utilizing a rotary evaporator at 40 °C under reduced pressure to concentrate the extract.

### 2.2 Chemicals

The chemicals were purchased from Sigma-Aldrich (Darmstadt, Germany). They were: 2,2′-azino-bis(3-ethylbenzothiazoline-6-sulphonic acid (ABTS), 1,1-diphenyl-2-picrylhydrazyl (DPPH), gallic acid, rutin, caffeic acid, electric eel acetylcholinesterase (AChE) (type-VI-S, EC 3.1.1.7), horse serum butyrylcholinesterase (BChE) (EC 3.1.1.8), galantamine, acetylthiocholine iodide (ATChI), butyrylthiocholine chloride (BTChI) 5,5-dithio-bis(2-nitrobenzoic) acid (DTNB), tyrosinase (EC1.14.18.1, mushroom), glucosidase (EC. 3.2.1.20, from *Saccharomyces cerevisiae*), amylase (EC. 3.2.1.1, from porcine pancreas), sodium carbonate, Folin–Ciocalteu reagent, hydrochloric acid, trolox, ethylenediaminetetraacetate (EDTA), neocuproine, cupric chloride, ammonium acetate, ferric chloride, 2,4,6-tris(2-pyridyl)-*s*-triazine (TPTZ), ammonium molybdate, ferrozine, ferrous sulphate hexahydrate, kojic acid and acarbose. All chemicals were of analytical grade.

#### 2.2.1 Determination of total phenolic and total flavonoid contents

To determine the Total Phenolic Content (TPC) of *R. caesius* root extracts, the Folin–Ciocalteu (FC) assay was employed.^14^ The resulting values were calculated using a gallic acid calibration curve and reported as mg GAE per g extract. For the assessment of Total Flavonoid Contents (TFC), an aluminum-complexation-based protocol was followed,^15^ in which the final concentrations were measured relative to rutin and expressed as mg RE per g extract. Calibration curves (Fig. S1) are given in the supplemental materials.

#### 2.2.2 UHPLC-MS/MS profiling of polyphenolic constituents

The detailed polyphenolic identification was achieved using a Dionex Ultimate 3000 UHPLC system equipped with DAD detector and TSQ Quantum Access Max triple-quadrupole mass spectrometer (ThermoFisher Scientific, Basel, Switzerland). The elution was carried out at 40 °C on a Syncronis C18 column (100 × 2.1 mm, 1.7 µm particle size), in accordance with the protocols of.^16^ The mobile phase consisted of (A) water + 0.1% formic acid (v/v), and (B) 100% acetonitrile (MS grade), which were applied in the following gradient elution: 5% B in the first 2.0 min, 2.0–14.0 min 5–95% B, 14.0–14.2 min from 95% to 5% B, and 5% B until the 20th min. The wavelength was set on 254 nm. The flow rate was set to 0.3 mL min^−1^ and the injection volume was 5 µL. The MS experiments were recorded in negative ionization mode. Final quantification, expressed in mg kg^−1^, was performed using Xcalibur software and a suite of phenolic standards to ensure precise annotation of the extracts.^17^

The phenolics were identified and quantified by direct comparison with commercial standards, according to the corresponding spectral characteristics: molecular ion, MS/MS spectra, characteristic fragmentation, and characteristic retention time. Validation parameters (retention times, precursor and product ions with specified collision energies, calibration range, equation parameters, determination coefficient (*R*^2^), LOD (limit of detection), and LOQ (limit of quantification)) of used UHPLC-MS/MS method are given in the Table S1. All compounds from Table S1 were supplied by Sigma-Aldrich (Steinheim, Germany). LOD (limit of detection) and LOQ (limit of quantitation) are calculated in excel using a calibration curve (linear regression) based on the residual standard deviation (*σ*) and slope (*S*) of the curve. The formulas are: LOD = (3 × *σ*)/*S* and LOQ = (10 × *σ*)/*S*. Very good linearity was shown for each compound with a coefficient of determination (*R*^2^) over 0.99.

#### 2.2.3 Antioxidant activity assays

To provide a comprehensive antioxidant profile, multiple *in vitro* models were utilized. The reducing power was assessed *via* Ferric Reducing Antioxidant Power (FRAP)^18^ and Cupric Ion Reducing Antioxidant Capacity (CUPRAC)^19^ methods, while scavenging effects were tested against 2,2-diphenyl-1-picrylhydrazyl (DPPH)^20^ and 2,2′-azino-bis(3-ethylbenzothiazoline-6-sulfonic acid) (ABTS) radicals.^21^ For the FRAP procedure, extracts were combined with a mixture of acetate buffer, TPTZ, and FeCl_3_·6H_2_O in a 10 : 1 : 1 ratio. In the CUPRAC assay, the reaction with 10 mM CuCl_2_ and 7.5 mM neocuproine was performed in ammonium acetate buffer (pH 7.0). Additionally, the phosphomolybdenum assay employed a reagent containing 0.6 M H_2_SO_4_, 28 mMNa_3_PO_4_, and 4 mM (NH_4_)_2_MoO_4_ to evaluate overall capacity at 695 nm.^22^ Metal sequestration was evaluated using a standard chelation assay, with findings expressed as mg EDTAE per g.^23^ All spectrophotometric measurements were validated against appropriate blanks and trolox standards.^24^

#### 2.2.4 Enzyme inhibitory activity

To evaluate biological activity, the root extracts were tested against various enzymes following standardized protocols. Cholinesterase inhibition was determined by tracking absorbance changes at 412 nm after initiating the reaction with acetylthiocholine or butyrylthiocholine.^25^ Similarly, tyrosinase inhibition was evaluated by recording the absorbance of the reaction mixture at 475 nm in the presence of l-DOPA.^26^ The anti-diabetic potential was examined through a-amylase and a-glucosidase assays, employing starch and pNPG as substrates, respectively.^27^ Post-reaction absorbance readings were taken at 540 nm and 405 nm after stopping the enzymatic activity with sodium carbonate. Results were validated using galantamine, kojic acid, and acarbose as positive benchmarks for the specific enzymes tested.

#### 2.2.5 Antimicrobial and antifungal activity

The antimicrobial efficacy of the root extracts was screened using a serial dilution method in 96-well plates, following the guidelines of the Clinical and Laboratory Standards Institute (CLSI). The following Gram-negative bacteria: *Escherichia coli* (ATCC 35210), *Salmonella typhimurium* (ATCC 13311), *Enterobacter cloacae* (ATCC 35030) and *Pseudomonas aeruginosa* (ATCC 27853) as well as Gram-positive bacteria: *Staphylococcus aureus* (ATCC 11632) *Bacillus cereus* (clinical isolate) *Micrococcus luteus* (ATCC 10240) were used. The organisms were obtained from the Mycological Laboratory, Department of Plant Physiology, Institute for Biological Research” Siniša Stankovic” – National Institute of Republic of Serbia, Belgrade, Serbia.

The antifungal activity of investigated of the root extracts was tested on strains obtained from the Mycological laboratory, Department of Plant Physiology, Institute for Biological Research “Sinisa Stankovic” National Institute of Republic of Serbia, Belgrade, Serbia. The following fungi *Aspergillus fumigatus* (ATCC 204305), *Aspergillus niger* (ATCC 6275), *Aspergillus versicolor* (ATCC 11730), *Aspergillus flavus* (ATCC 9170), *Trichoderma viride* (IAM 5061), *Penicillium ochrochloron* (ATCC 9112), *Penicillium verrucosum* var. *cyclopium* (food isolate) and *Penicillium funiculosum* (ATCC 36839) and were tested.

Microbial cultures were standardized to 1 × 10^5^ CFU mL^−1^ before being added to the wells. The extracts were diluted in specific media (Mueller–Hinton/Malt broth medium or RPMI-1640) to reach final concentrations between 1.95 and 1000 µg mL^−1^. After the required incubation periods, the presence of viable microorganisms was detected using resazurin dye. Commercial antibiotics ampicillin and streptomycin such as fungicide bifonazole and ketoconazole (Sigma-Aldrich, China) were used as positive controls.^17^

#### 2.2.6 Molecular docking

Following geometry optimization *via* DFT, the lowest-energy conformations of seven compounds were selected for molecular docking analyses carried out with the PLANTS docking software.^28^ The three-dimensional crystallographic structures of the target proteins—acetylcholinesterase (AChE, PDB ID: 4EY5),^29^ butyrylcholinesterase (BChE, PDB ID: 4TPK),^30^ tyrosinase (TYR, PDB ID: 1WX2),^31^ amylase (AML, PDB ID: 9FZ3),^32^ and glucosidase (GLC, PDB ID: 2RGL)^33^—were obtained from the RCSB Protein Data Bank.^34^ Docking binding sites were defined based on the positions of co-crystallized ligands within each protein structure, and the centers of these binding pockets were determined using the coordinates of the native ligands. Accordingly, the grid centers were assigned as follows: *x* = −9.45, *y* = −43.66, *z* = 31.87 for AChE; *x* = 4.02, *y* = 10.76, *z* = 13.86 for BChE; *x* = −12.27, *y* = −13.17, *z* = 18.07 for TYR; *x* = 31.10, *y* = 55.65, *z* = 31.02 for AML; and *x* = 3.79, *y* = 2.09, *z* = 35.90 for GLC. To validate the docking protocol, a redocking procedure was performed using the co-crystallized native ligand of each target protein, confirming that the selected docking parameters were capable of accurately reproducing the experimentally observed binding poses. Protein preparation was carried out using the Dock Prep tool within UCSF Chimera.^35^ This process involved removing crystallographic water molecules and non-standard residues, adding hydrogen atoms, and assigning appropriate partial charges. The ligand structures optimized at the DFT level were subsequently converted into.mol2 format and used as input for molecular docking. For each ligand, the binding pose with the best score according to the PLANTS scoring function was selected as the most favorable conformation. The resulting protein–ligand complexes were visualized using PyMOL^36^ and further examined with BIOVIA Discovery Studio Visualizer.^37^ In addition, two-dimensional interaction diagrams were generated to characterize key non-covalent interactions within the binding site, including hydrogen bonding, hydrophobic interactions, and pi–pi stacking.

#### 2.2.7 Molecular dynamics simulations and free energy calculations

Molecular dynamics (MD) simulations were carried out using GROMACS 2024.1 (ref. 38) to examine the stability and dynamic behavior of the docked protein–ligand complexes. For each target protein, the two ligands with the most favorable docking scores were selected, and their best binding conformations were used as the initial structures for the simulations. The proteins were described using the AMBER ff99SB force field,^39^ while ligand parameters were generated with ACPYPE.^40^ Each system was embedded in a dodecahedron simulation box with a minimum distance of 1.0 nm from the box edges and solvated using the TIP3P water model. Counterions were added to neutralize the overall charge of the system. Energy minimization was first performed using the steepest descent algorithm, followed by equilibration steps of 250 ps under both NVT and NPT ensembles with positional restraints applied to the heavy atoms. The system temperature (300 K) and pressure (1 bar) were maintained using the V-rescale thermostat and the Parrinello–Rahman barostat, respectively. Long-range electrostatic interactions were treated with the Particle Mesh Ewald (PME) approach, and a cutoff distance of 1.4 nm was applied for both electrostatic and van der Waals interactions. After equilibration, 100 ns production MD simulations were conducted for each complex. Trajectory analyses were performed using GROMACS 2024.1 to evaluate structural stability and flexibility through root-mean-square deviation (RMSD), root-mean-square fluctuation (RMSF), radius of gyration (*R*_g_), and hydrogen bond analysis.

Binding free energy was estimated using the gmx_MMPBSA^41^ based on the MM-PBSA approach. Energy values were averaged over multiple snapshots extracted from the full MD trajectories to ensure adequate conformational sampling. Furthermore, energy decomposition analyses were performed to quantify the individual contributions of van der Waals, electrostatic, polar solvation, and non-polar solvation terms to the overall binding affinity.

#### 2.2.8 Density functional theory (DFT) calculations

Based on the LC-MS analysis, seven *R. caesius* compounds that were consistently identified in both the aqueous and ethanol extracts were selected for DFT calculations and molecular docking. Within the theoretical framework of this study, seven main components of the investigated *R. caesius* plant extra were examined comprehensively. Ellagic acid, eriodictyol, isorhamnetin, isorhamnetin 3-*O*-rutinoside, kaempferol 3-*O*-glucoside, quercetin 3-*O*-glucoside, and rutin compounds were identified as the dominant bioactive molecules and subsequently selected for in-depth quantum chemical analysis. The molecular structures were fully optimized using the ORCA 4.2.1 computational chemistry package^42^ within the framework of Density Functional Theory (DFT). Calculations were performed employing the B3LYP hybrid exchange–correlation functional^43^ together with the 3-21G basis set.^44^ All geometry optimizations were carried out in the gas phase, and no symmetry constraints were applied during the optimization process. After the optimization step, the electronic properties and reactivity patterns of the molecules were analyzed through conceptual DFT descriptors obtained from frontier molecular orbital energies. In the scope of the Conceptual Density Functional Theory, chemical reactivity descriptors provide a comprehensive depiction of the electronic structure, stability, and chemical reactivity behavior of the investigated systems such as ionization potential (*I*), electron affinity (*A*), electronegativity (*χ*), global hardness (*η*), global softness (*σ*), chemical potential (*µ*), and the electrophilicity index (*ω*) are predicted from the following equations.^45^*I* = *E*_HOMO_*A* = −*E*_LUMO_
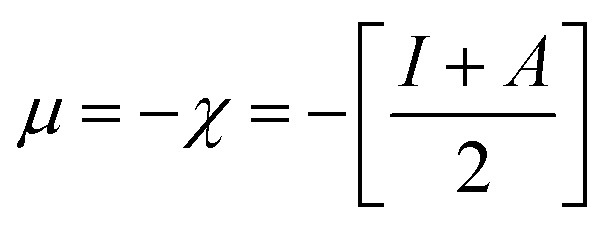

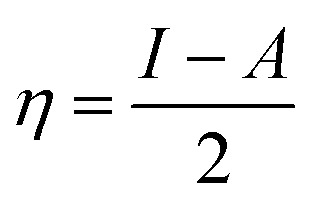
*σ* = 1/*η**ω* = *χ*^2^/2*η* = *µ*^2^/2*η*

The global electrophilicity index (*ω*) quantifies the degree to which a molecule becomes stabilized upon gaining additional electronic charge and thus provides a numerical measure of its propensity to act as an electron acceptor. Molecules exhibiting high *ω* values are typically strong electrophiles, whereas lower *ω* values are more commonly associated with nucleophilic behavior. Within the conceptual DFT framework introduced by Parr, this descriptor is defined as a function of electronegativity (*χ*) and chemical hardness (*η*).^46^ On the other hand, global softness (*σ*) reflects how readily a molecule's electron distribution can be distorted by external influences. As the reciprocal of chemical hardness (*η*), it complements the information provided by hardness in assessing molecular reactivity and stability. Higher *σ* values indicate greater polarizability and an increased likelihood of engaging in chemical interactions, thereby signifying enhanced reactivity.

## 3 Results and discussion

### 3.1 TPC and TFC analysis

**Fig. 1:**
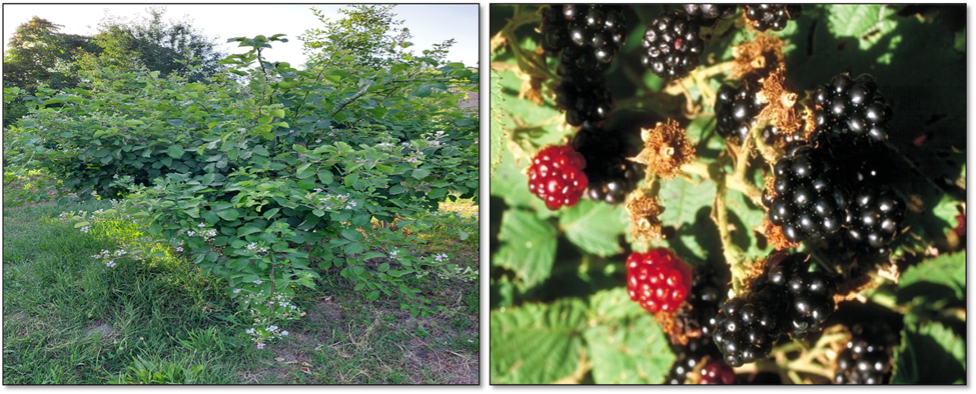
Aerial parts of *Rubus caesius*.

The extraction yield of the ethanolic extract (23.43 ± 1.08%) was significantly higher than that of the infusion extract (18.57 ± 0.67%) (*p* < 0.01) (Table 1). Table 1 also summarizes the TPC and TFC of *Rubus caesius* root extracts prepared by infusion and ethanol extraction. The TPC for the root extracted through infusion was found to be 110.48 ± 0.79 mg GAE per g, while the TFC was found to be 3.92 ± 0.33 mg RE per g. On the other hand, the TPC for the RCR extracted through ethanol was found to be 101.01 ± 1.73 mg GAE per g, while the TFC was found to be 5.54 ± 0.035 mg RE per g. These values suggest that both infusion and ethanol methods can be effective in extracting phenolic and flavonoid compounds from *R. caesius* root. The infusion method yielded higher TPC, while the ethanol method yielded higher TFC. These results provide insights into the potential health benefits of using *R. caesius* root extracts across various applications, including functional foods and natural remedies (Fig. 1). Phytochemical analyses confirm that *R. caesius* contains flavonoids, ellagic acid-related phenolics, and tannins, despite most published research on the species focusing on leaves and stems.^12^ In this regard, the current RCR extracts with high TPC, but relatively low TFC most likely reflect a phenolic profile dominated by non-flavonoid phenolics (such as tannins/ellagic derivatives), with flavonoids comprising a smaller portion of the total phenolic pool. This interpretation is consistent with the chemical classes reported for *R. caesius* and with more general summaries of phenolics found across *Rubus* taxa.^12^

**Table 1:** Extraction yield%, total phenolic, flavonoid infusion and ethanol extract of RCR^a^

Extracts	Extraction yield%	TPC (mg GAE per g)	TFC (mg RE per g)
Infusion	18.57 ± 0.67^a^	110.48 ± 0.79^a^	3.92 ± 0.33^b^
Ethanol	23.43 ± 1.08^b^	101.01 ± 1.73^b^	5.54 ± 0.04^a^

a Values are reported as mean ± SD of three parallel measurements. TPC: total phenolic content; TFC: total flavonoid content; GAE: gallic acid equivalent; RE: rutin equivalent.

### 3.2 LC-MS analysis

The quantitative LC-MS results revealed that *Rubus caesius* root is particularly rich in ellagic acid and flavonoid glycosides, with clear differences between infusion and ethanol extracts (Table 2). Among all detected compounds, ellagic acid was the dominant phenolic in both extracts, reaching 1658.88 mg kg^−1^ in the infusion extract and 1232.54 mg kg^−1^ in the ethanol extract. This high concentration is consistent with previous studies reporting that *Rubus* species are characterized by high levels of ellagitannins and their hydrolysis product, ellagic acid, which constitute the major phenolic class in roots and other woody tissues.^11,12,47^ The higher level in the infusion extract suggests that water is more effective for recovering hydrolyzable tannin derivatives and highly polar phenolics. Flavonoid glycosides constituted the second major group of compounds. Rutin (36.77 and 29.65 mg kg^−1^) and quercetin-3-*O*-glucoside (19.57 and 28.41 mg kg^−1^) were among the predominant flavonoids, together with isorhamnetin-3-*O*-rutinoside (141.38 and 131.98 mg kg^−1^) (Table 2). These findings are in agreement with literature reports indicating that quercetin and isorhamnetin glycosides are typical flavonols in *Rubus* species.^11,47^ The slightly higher levels of quercetin glycoside and several minor flavonoids in the ethanol extract confirm that hydroalcoholic solvents improve the extraction of moderately polar flavonoids. This trend also corresponds with the higher total flavonoid content (TFC) observed in the ethanol extract (5.54 mg RE per g) relative to the infusion extract (3.92 mg RE per g). The combined LC-MS and spectrophotometric results therefore demonstrate a clear solvent-dependent selectivity, where ethanol favors the recovery of flavonoid-related metabolites, whereas water enhances the extraction of ellagitannin-derived phenolics. While the targeted LC-MS method was optimized specifically for a certain number of compounds (Table 2), the broader DAD screening at 254 nm highlights the presence of additional co-extracted matrix constituents, which can be seen in Fig. S2.

**Table 2:** Bioactive component contents (µg per g of dry weight extract) in infusion and ethanol extracts of RCR. Three separate analyses were conducted. (NF: not found)

Compounds	Aqueous extract (µg g^−1^)	Ethanol extract (µg g^−1^)
3-*O*-Caffeoylquinic_acid	0.50 ± 0.01	0.57 ± 0.02
5-*O*-Caffeoylquinic_acid	NF	5.82 ± 0.08
Caffeic_acid	0.84 ± 0.01	0.85 ± 0.04
Rutin	36.77 ± 0.50	29.65 ± 0.53
Vitexin	1.00 ± 0.02	1.08 ± 0.02
*p*-Coumaric_acid	NF	19.67 ± 1.23
Quercetin_3-*O*-glucoside	19.57 ± 0.69	28.41 ± 0.30
Ellagic_acid	1658.88 ± 11.10	1232.54 ± 25.22
Isorhamnetin_3-*O*-rutinoside	141.38 ± 4.89	131.98 ± 4.15
Kaempferol_3-*O*-glucoside	5.25 ± 0.08	7.15 ± 0.20
Eriodictyol	1.20 ± 0.01	1.90 ± 0.07
Naringenin	1.05 ± 0.02	0.98 ± 0.07
Hispidulin	NF	0.28 ± 0.01
Isorhamnetin	9.34 ± 0.12	NF

Phenolic acids showed solvent-dependent selectivity. 5-*O*-caffeoylquinic acid and *p*-coumaric acid were detected only in the ethanol extract (5.82 and 19.67 mg kg^−1^, respectively), whereas isorhamnetin aglycone was observed only in the aqueous extract. The presence of chlorogenic acid derivatives (caffeoylquinic acids) and hydroxycinnamic acids is consistent with previous phytochemical studies of *Rubus* taxa.^48^ The preferential extraction of these compounds in ethanol may be attributed to their intermediate polarity and stronger interactions with organic solvents. Moreover, the selective recovery of hydroxycinnamic acids in the ethanol extract may partially explain the increased flavonoid-associated antioxidant behavior of this extract, since these compounds are known to exhibit strong radical-scavenging and metal-chelating activities.

Minor flavonoids such as vitexin, kaempferol-3-*O*-glucoside, eriodictyol, naringenin, and hispidulin were detected at low concentrations (<10 mg kg^−1^), indicating that the phenolic profile of RCR is dominated by ellagic acid and flavonol glycosides rather than flavanones or flavones. Briefly, the compositional pattern supports the previously observed high total phenolic content and is consistent with the strong antioxidant potential reported for *Rubus* extracts, as ellagic acid and quercetin derivatives have previously been associated with antioxidant activity.^11,49^ The predominance of ellagic acid, together with quercetin and isorhamnetin derivatives, suggests possible synergistic antioxidant interactions among these compounds, potentially enhancing the overall bioactivity of the extracts beyond the contributions of individual metabolites.

In summary, the results confirm that RCR is a tannin-rich phenolic source dominated by ellagic acid, with ethanol extraction favoring flavonoids and hydroxycinnamic acids, while infusion extraction enhances the recovery of highly polar ellagitannin-derived compounds. This solvent-dependent distribution is consistent with the phytochemical characteristics reported for the genus and supports the plant's potential functional and antioxidant value. The strong agreement between LC-MS profiling and spectrophotometric TPC/TFC analyses further supports the reliability of the phytochemical characterization and demonstrates that the choice of extraction solvent decisively influences the qualitative and quantitative composition of the bioactive metabolites obtained.

### 3.3 Antioxidant properties

The antioxidant capacity of *R. caesius* root showed clear solvent-dependent differences across the applied assays (Table 3). Taken together, the infusion extract demonstrated stronger antioxidant performance than the ethanol extract in most tests, including PBD (2.84 ± 0.18 *vs.* 2.04 ± 0.14 mmol TE per g), DPPH (469.15 ± 4.74 *vs.* 376.59 ± 3.99 mg TE per g), CUPRAC (906.45 ± 11 *vs.* 791.93 ± 20.31 mg TE per g), FRAP (561.24 ± 11.87 *vs.* 425.52 ± 16 mg TE per g), and metal chelating activity (24.51 ± 0.17 *vs.* 8.32 ± 0.34 mg EDTAE per g). In contrast, the ethanol extract exhibited slightly higher ABTS radical scavenging activity (781.10 ± 7.13 *vs.* 752.23 ± 0.69 mg TE per g). These findings indicate that aqueous extraction enhances the recovery of compounds associated with reducing power and metal chelation, whereas ethanol may improve the extraction of certain radical-scavenging constituents detectable by the ABTS assay. This solvent-dependent antioxidant behavior strongly correlates with the LC-MS phytochemical profile, with the aqueous extract containing substantially higher levels of ellagic acid (1658.88 mg kg^−1^) and detectable amounts of isorhamnetin, whereas the ethanol extract was comparatively enriched in flavonoid glycosides including quercetin-3-*O*-glucoside and kaempferol-3-*O*-glucoside.

**Table 3:** Comparative antioxidant capacity of RCR infusion and ethanolic extracts^a^

Extracts	PBD (mmol TE per g)	DPPH (mg TE per g)	ABTS (mg TE per g)	CUPRAC (mg TE per g)	FRAP (mg TE per g)	MCA (mg EDTAE per g)
Infusion	2.84 ± 0.18^a^	469.15 ± 4.74^a^	752.23 ± 0.69^b^	906.45 ± 11.00^a^	561.24 ± 11.87^a^	24.51 ± 0.17^a^
Ethanol	2.04 ± 0.14^b^	376.59 ± 3.99^b^	781.10 ± 7.13^a^	791.93 ± 20.31^b^	425.52 ± 16.00^b^	8.32 ± 0.34^b^

a Values are reported as mean ± SD of three parallel measurements. TE: trolox equivalent; PBD: phosphomolybdenum; MCA: metal chelating; EDTAE: EDTA equivalent.

The stronger antioxidant activity of the infusion extract is consistent with its higher total phenolic content and the predominance of highly polar phenolics such as ellagic acid and tannin-derived compounds. Phenolic acids and ellagitannins are known to contribute significantly to electron-donating ability, ferric-reducing capacity, and transition-metal chelation, which may partly explain their superior performance in FRAP, CUPRAC, and MCA assays.^49^ The exceptionally high concentration of ellagic acid in the aqueous extract may have contributed to in the observed antioxidant activity, as ellagic acid possesses multiple hydroxyl groups that can donate electrons and stabilize reactive oxygen species. In addition, tannin-derived compounds are known for their strong affinity for transition metals, which may explain the markedly higher metal-chelating activity of the infusion extract. The slightly higher ABTS activity in the ethanol extract may be attributed to improved extraction of moderately polar flavonoids, such as quercetin derivatives, which are effective radical scavengers in both hydrophilic and lipophilic systems.^19^

Comparatively, previous studies on *Rubus* species have reported strong antioxidant activities closely associated with high levels of ellagic acid, ellagitannins, and flavonol glycosides.^11,12^ The antioxidant values obtained in this study are within the range reported for phenolic-rich *Rubus* extracts and suggest that roots, like aerial parts, can serve as a potent source of natural antioxidants. The agreement between antioxidant assays and chemical profiling further strengthens the conclusion that the antioxidant potential of *R. caesius* root may be largely associated with its phenolic composition. Specifically, the dominance of ellagic acid together with rutin, isorhamnetin derivatives, and quercetin glycosides suggests that synergistic interactions among these compounds may contribute to the broad-spectrum antioxidant activity observed across multiple assay systems.^11^

To summarize, the results demonstrate that RCR exhibits strong, multi-mechanistic antioxidant activity, including radical scavenging, reducing power, and metal chelation. The observed solvent-dependent behavior is consistent with the phenolic composition profile and with previous reports describing the antioxidant properties of phenolic-rich extracts from the genus *Rubus*.^50,51^ Overall, the combined phytochemical and antioxidant findings indicate that aqueous extraction is particularly effective for recovering tannin-derived antioxidant compounds, whereas ethanol extraction preferentially concentrates flavonoid-related radical scavengers. This complementary phytochemical distribution highlights the potential of *R. caesius* root as a versatile natural antioxidant source with possible applications in pharmaceutical, nutraceutical, and functional food formulations.

### 3.4 Enzyme inhibitory effects

The enzyme inhibition results show that the ethanolic extract of *R. caesius* root exhibited higher activity than the infusion (Table 4), particularly for AChE (2.98 mg GALAE per g), BChE (1.76 mg GALAE per g), α-amylase (0.30 mmol ACAE per g), and tyrosinase (81.25 mg KAE per g), while α-glucosidase inhibition remained similar for both extracts (1.05 mmol ACAE per g). This pattern is consistent with previous studies showing that organic solvents extract phenolic compounds more efficiently than water, resulting in stronger enzyme inhibitory effects.^52,53^ The LC-MS analysis supports these findings, as the ethanolic extract contained higher levels of flavonoid-related constituents, including quercetin-3-*O*-glucoside, kaempferol-3-*O*-glucoside, and hydroxycinnamic acid derivatives, which are widely associated with enzyme-inhibitory potential. In addition, the higher total flavonoid content (TFC) of the ethanolic extract may partly explain its superior inhibitory performance against several target enzymes.

**Table 4:** Comparative enzyme inhibition profiles of *Rubus caesius* root infusion and ethanolic extracts^a^

Extracts	AChE (mg GALAE per g)	BChE (mg GALAE per g)	α-Amylase (mmol ACAE per g)	α-Glucosidase (mmol ACAE per g)	Tyrosinase (mg KAE per g)
Infusion	1.40 ± 0.07^b^	na	0.06 ± 0.01^b^	1.05 ± 0.01^a^	34.35 ± 1.28^b^
Ethanol	2.98 ± 0.01^a^	1.76 ± 0.16	0.30 ± 0.01^a^	1.05 ± 0.01^a^	81.25 ± 0.64^a^

a Values are reported as mean ± SD of three parallel measurements. GALAE: galanthamine equivalent; ACAE: acarbose equivalent; KAE: kojic acid equivalent.; na: not active.

The moderate cholinesterase inhibition observed in the ethanolic extract is consistent with reports in other *Rubus* species, where AChE and BChE inhibition has been associated with ellagic acid, flavonoids, and proanthocyanidins, compounds known for their neuroprotective potential.^54,55^ Although the aqueous extract contained higher amounts of ellagic acid, the stronger cholinesterase inhibition of the ethanolic extract suggests that flavonoid glycosides and moderately polar phenolics may play a more important role in enzyme binding and inhibition. Synergistic interactions among quercetin derivatives, rutin, and other phenolics may also contribute to enhanced inhibitory activity.

Regarding antidiabetic enzymes, the tested extract showed mild to moderate inhibition of α-amylase and α-glucosidase, consistent with previous studies reporting that berry polyphenols exert moderate inhibitory effects on carbohydrate-hydrolyzing enzymes, therewith contributing to postprandial glycemic regulation.^56^ The slightly stronger α-amylase inhibition observed for the ethanolic extract may be associated with its enrichment in flavonoids and phenolic acids, particularly quercetin glycosides and *p*-coumaric acid, which have previously been linked to mechanisms of carbohydrate enzyme inhibition. The similar α-glucosidase inhibition values observed for both extracts suggest that polar tannin-derived compounds and moderately polar flavonoids may contribute to this activity.

Notably, the strong tyrosinase inhibitory activity observed for the tested extract is comparable to phenolic-rich plant extracts reported in the literature. Polyphenols including flavonoids and phenolic acids are known to inhibit tyrosinase by interacting with the enzyme's copper-containing active site.^56^ The significantly higher tyrosinase inhibition exhibited by the ethanolic extract may related to its greater abundance of flavonoid glycosides and hydroxycinnamic acid derivatives, which have been reported to be effective metal-chelating and copper-binding agents. This observation also correlates with the extract's antioxidant profile, since compounds capable of radical-scavenging and reducing activities commonly exhibit parallel tyrosinase-inhibitory effects. This suggests that the tested extract could be considered a promising natural candidate for cosmetic or food applications aimed at controlling hyperpigmentation or enzymatic browning. Taken together, the results are consistent with the literature on *Rubus* species, confirming that phenolic-rich extracts exhibit multifunctional enzyme-inhibitory properties and bringing out the root as a relatively underexplored yet biologically valuable plant part.^51,57^ While aqueous extraction favored tannin-derived antioxidant phenolics such as ellagic acid, whereas ethanolic extraction preferentially enriched flavonoid-associated compounds that may contribute to enzyme inhibitory activity. This complementary distribution of bioactive metabolites stresses the therapeutic and functional potential of *R. caesius* root as a natural source of multifunctional phytochemicals.

### 3.5 Antibacterial activity

The Table 5 demonstrated that *R. caesius* root extracts possess broad-spectrum but moderate antimicrobial activity, with MIC values ranging between 0.5–3.0 mg mL^−1^ for the infusion and 0.5–2.0 mg mL^−1^ for the ethanolic extract, while MBC values varied from 1.0 to 4.0 mg mL^−1^. In general, the ethanolic extract showed slightly stronger activity against some strains, particularly *Micrococcus luteus*, which may be attributed to the higher extraction efficiency of phenolic compounds, flavonoids, and tannins in organic solvents. These secondary metabolites may contribute to the antimicrobial activity of *Rubus* species.^1,12,57^ This observation is also supported by the phytochemical analysis, in which the ethanolic extract exhibited relatively higher levels of flavonoid glycosides, such as quercetin-3-*O*-glucoside and kaempferol-3-*O*-glucoside, which have been previously associated with membrane disruption, inhibition of microbial enzymes, and induction of oxidative stress in bacterial cells.^58,59^

**Table 5:** Comparative antibacterial activity of *Rubus caesius* root infusion and ethanolic extracts^a^

		S.a.	B.c.	L.m.	M.l.	P.ae.	E.c.	S.t.	En.cl.
Infusion	MIC	0.5	0.5	0.5	3.0	1.5	0.5	1.0	0.5
	MBC	1.0	1.0	1.0	4.0	2.0	1.0	2.0	1.0
Ethanol	MIC	0.5	0.5	1.0	2.0	1.5	1.0	1.0	1.0
	MBC	1.0	1.0	2.0	4.0	2.0	2.0	2.0	2.0
Streptomycin	MIC	0.100	0.025	0.150	0.050	0.100	0.100	0.100	0.025
	MBC	0.200	0.050	0.300	0.100	0.200	0.200	0.200	0.050
Ampicillin	MIC	0.100	0.100	0.150	0.100	0.300	0.150	0.100	0.100
	MBC	0.150	0.150	0.500	0.150	0.500	0.200	0.200	0.150

a Minimum inhibitory concentration (MIC) and minimum bactericidal concentration (MBC) values. S.a.: *Staphylococcus aureus*; B.c.: *Bacillus cereus*; M.l.: *Micrococcus luteus*; P.a.: *Pseudomonas aeruginosa*; E.c.: *Escherichia coli*; S.t.: *Salmonella typhimurium*; En.cl: *Enterobacter cloacae*.

The extracts were more effective against Gram-positive bacteria (*Staphylococcus aureus*, *Bacillus cereus*, *Listeria monocytogenes*) than Gram-negative strains, whereas relatively higher MIC values were observed for *Pseudomonas aeruginosa*. This pattern is consistent with previous reports on *Rubus* extracts and other phenolic-rich plant materials, in which the outer membrane of Gram-negative bacteria limits the penetration of polyphenolic compounds and reduces susceptibility.^1,57^ The comparatively lower susceptibility of *P. aeruginosa* may additionally be related to its strong intrinsic resistance mechanisms, including efflux pumps and reduced membrane permeability.^60^ In contrast, Gram-positive bacteria possess a less complex cell envelope, allowing phenolic constituents to interact more efficiently with the cell membrane and intracellular targets.^61^

Comparison of MIC and MBC values indicates that, for several microorganisms, the MBC/MIC ratio was close to 2, suggesting a bactericidal effect, while higher ratios observed for certain strains point to predominantly bacteriostatic action. Similar activity levels (generally within the range of 0.5–5 mg mL^−1^) have been reported for *R. caesius* aerial parts and other *Rubus* species, although antimicrobial potency is known to depend strongly on plant part, extraction solvent, and phytochemical composition.^1,12,52,62^ Ellagic acid, rutin, and other flavonoids detected by LC-MS may collectively contribute to this activity through synergistic mechanisms involving protein binding, membrane destabilization, and interference with bacterial metabolic pathways.^58,63^

Although the activity of the root extracts was markedly lower than that of standard antibiotics (streptomycin and ampicillin), the observed inhibitory effects are typical for crude plant extracts and support the role of *R. caesius* roots as a natural source of bioactive phenolic compounds with moderate antibacterial potential. Furthermore, the coexistence of antioxidant and antibacterial activities suggests that the extracts may exert multifunctional biological effects that may be associated with their phenolic-rich composition.^59^ These findings highlight *R. caesius* root as an underexplored plant material with potential relevance for future pharmaceutical, nutraceutical, or natural preservative applications.

### 3.6 Antifungal activity

Table 6 provides antifungal results for *R. caesius* root extracts demonstrating moderate activity against a panel of filamentous fungi, including *Aspergillus* spp. (A.f. likely *A. fumigatus*, A.n. *A. niger*, A.v. *A. versicolor*, A.fl. *A. flavus*), *Trichophyton* sp. (*T. viride*), and *Penicillium* spp. (P.f. likely *P. funiculosum*, P.o. possibly *P. ochro-chloron*, P.v.c. *P. verrucosum* var. *cyclopium* or similar). MIC values range from 1.5–4.0 mg mL^−1^ for the infusion (strongest against A.v. at 1.5 mg mL^−1^) and 1.5–3.0 mg mL^−1^ for the ethanol extract (strongest against A.fl. at 1.5 mg mL^−1^). MFC values are mostly 4.0–8.0 mg mL^−1^, with MFC/MIC ratios of 2–5 (often ∼4), indicating primarily fungistatic effects, though some fungicidal potential at higher concentrations (*e.g.*, against A.v. and A.n. with infusion). Both extracts show comparable broad-spectrum moderate activity, with ethanol slightly more effective against certain *Aspergillus* spp. (lower MIC for A.n. and A.fl.) and infusion stronger against A.v. The weakest activity is observed against P.v.c. (MIC 3.0–4.0 mg mL^−1^). These crude extracts are significantly less potent than reference antifungals bifonazole (MIC 0.100–0.200 mg mL^−1^, MFC 0.200–0.250 mg mL^−1^) and ketoconazole (MIC 0.200–1.000 mg mL^−1^, MFC 0.300–1.500 mg mL^−1^), which is typical for unpurified plant materials *versus* synthetic drugs. The activity may be associated with polyphenolic compounds (*e.g.*, ellagitannins, flavonoids, phenolic acids) that are common in *Rubus* species and that disrupt fungal membranes, inhibit enzymes, or interfere with ergosterol biosynthesis.^1,5,64^ The LC-MS analysis is consistent with this interpretation, as the extracts contained high levels of ellagic acid, together with rutin, quercetin glycosides, and isorhamnetin derivatives, compounds previously associated with alterations in membrane permeability and the induction of oxidative stress in fungal cells.^58,59^

**Table 6:** Comparative antifungal activity of *Rubus caesius* root infusion and ethanolic extracts^a^

		A.f.	A.n.	A.v.	A.fl.	T.v.	P.f.	P.o.	P.v.c.
Infusion	MIC	2.0	3.0	1.5	2.0	2.0	2.0	2.0	4.0
	MFC	4.0	4.0	4.0	4.0	4.0	4.0	8.0	8.0
Ethanol	MIC	2.0	2.0	3.0	1.5	3.0	3.0	3.0	3.0
	MFC	8.0	8.0	4.0	8.0	8.0	8.0	8.0	8.0
Bifonazole	MIC	0.150	0.150	0.100	0.150	0.150	0.200	0.200	0.100
	MFC	0.200	0.200	0.200	0.200	0.200	0.250	0.250	0.200
Ketoconazole	MIC	0.200	0.200	0.200	0.200	1.000	0.200	1.000	0.200
	MFC	0.500	0.500	0.500	0.500	1.500	0.500	1.500	0.300

a Minimum inhibitory concentration (MIC) and minimum fungicidal concentration (MFC) values. A.f.: *Aspregillus fumigatus*; A.n.: *Aspergillus niger*; A.v.: *Aspergillus versicolor*; A.fl.: *Aspergillus flavus*; T.v: *Trichoderma viride*; P.f.: *Penicillium funiculosum*; P.o.: *Penicillium ochrochloron*; P.v.c.: *Penicillium verrucosum* var. *cyclopium*.

Published literature on *Rubus caesius* antifungal activity remains very limited and almost exclusively focuses on leaves and stems, with no direct reports identified for root extracts or against the specific filamentous molds tested here (*Aspergillus*, *Penicillium*, *Trichophyton*).^1,5,64,65^ Studies on aerial parts (young leaves and stems) report weak to negligible antifungal effects, particularly against yeasts: water and ethanol extracts showed high MIC values (10–50 mg mL^−1^ or >50 mg mL^−1^) against *Candida albicans* and *C. parapsilosis*, far weaker than amphotericin B (0.002 mg mL^−1^), and were described as having “low activity” or “none significant” compared to standards. No meaningful activity against molds was highlighted in these works, which primarily emphasized antibacterial potency (*e.g.*, against *Clostridium* spp., *Enterococcus faecalis*, or MRSA strains with MICs often 0.16–5 mg mL^−1^ for leaves/stems). Related *Rubus* species (*e.g.*, *R. discolor* fruits or leaves) occasionally exhibit antifungal effects against molds such as *Trichoderma viride*, *Penicillium* spp., or *Aspergillus* in other contexts, but data are sparse and not directly comparable. Compared with previous findings, the root extracts evaluated in the present study appear to exhibit stronger inhibitory effects against filamentous fungi, particularly *Aspergillus* species. This difference may indicate that roots accumulate distinct phenolic metabolites or higher concentrations of antifungal constituents than aerial tissues. The predominance of ellagic acid and tannin-derived compounds in the roots may be particularly relevant, since tannins are known to form complexes with fungal cell wall proteins and membrane-associated enzymes.^66,67^

Related *Rubus* species (*e.g.*, *R. ulmifolius* fruits containing sanguiin H-6) exhibit fungistatic effects against *C. albicans* (MIC 5 mg mL; no fungicidal activity), consistent with moderate levels.^64^ The observed MICs (1.5–4.0 mg mL^−1^) against molds appear stronger than the yeast-targeted aerial-part data (>10 mg mL^−1^), suggesting that roots may concentrate relevant secondary metabolites differently or exhibit greater activity against filamentous fungi.^1,5,64^ The predominance of fungistatic rather than fungicidal effects in the current work may reflect the complex nature of crude extracts, where active constituents occur at suboptimal concentrations and may act synergistically rather than individually. Moreover, the stronger activity of the ethanolic extract against *A. flavus* and *A. niger* is consistent with its higher flavonoid content, suggesting that moderately polar flavonoids may contribute more effectively to antifungal activity against certain molds.^68^

In summary, these findings position *R. caesius* root extracts as a moderately effective natural option against common environmental and pathogenic molds (*Aspergillus* and *Penicillium* spp. in particular), outperforming the reported activity of aerial parts against yeasts. The combined phytochemical and biological results suggest that solvent polarity appears to influence antifungal performance through selective extraction of tannins, flavonoids, and phenolic acids. Although the extracts were less potent than commercial antifungals, their broad-spectrum fungistatic activity, together with strong antioxidant properties, highlights the roots as a promising and previously underexplored source of multifunctional bioactive compounds. Further fractionation and isolation of active constituents may help identify the compounds primarily responsible for the observed antifungal effects and improve potency toward pharmaceutical or food-preservation applications.^59,67^

### 3.7 Docking analysis

To gain deeper insight into the interaction capabilities of the selected seven compounds, molecular docking studies were performed using the PLANTS docking software against five target enzymes: acetylcholinesterase (AChE), butyrylcholinesterase (BChE), tyrosinase (TYR), amylase (AML), and glucosidase (GLC). The docking protocol was validated by re-docking the co-crystallized ligands into the binding sites of AChE, BChE, AML, and GLC using the same docking parameters. The resulting RMSD values were 0.190 Å for AChE, 1.742 Å for BChE, 1.008 Å for AML, and 1.058 Å for GLC, all of which were below the generally accepted validation threshold of 2.0 Å, confirming the reliability of the docking protocol. Re-docking validation was not performed for TYR because a peroxide molecule is present in the crystallographic binding site. The calculated docking scores (kcal mol^−1^) for each protein–ligand complex are presented in Table 7. The molecular docking analysis revealed a diverse range of binding affinities between the seven evaluated phytochemicals and the five target enzymes, with docking scores spanning from −33.14 to −106.18 kcal mol^−1^. Notably, rutin exhibited a remarkable preference for BChE, yielding the most potent interaction in the study with a score of −106.18 kcal mol^−1^, closely followed by quercetin 3-*O*-glucoside (−101.60 kcal mol^−1^) and isorhamnetin 3-*O*-rutinoside (−101.03 kcal mol^−1^). This suggests that the BChE active site is highly conducive to the structural profiles of these specific glycosides. In contrast, eriodictyol emerged as a particularly versatile multi-target candidate, demonstrating the highest stability against AChE at −96.51 kcal mol^−1^, while maintaining robust and consistent performance across AML, GLC, and TYR. Interestingly, while rutin displayed exceptional affinity for BChE, it showed the weakest interaction with AChE (−33.14 kcal mol^−1^), highlighting a significant degree of selectivity. Furthermore, for the GLC enzyme, both eriodictyol and quercetin 3-*O*-glucoside showed superior binding stability, whereas isorhamnetin 3-*O*-rutinoside exhibited a notable decline in affinity (−36.47 kcal mol^−1^) compared to its other targets.

**Table 7:** Docking results (kcal mol^−1^) for the seven most prominent compounds

Molecules/proteins	AChE (4EY5)	AML (9FZ3)	BChE (4TPK)	GLC (2RGL)	TYR (1WX2)
Ellagic acid	−81.20	−63.31	−86.82	−73.19	−76.05
Eriodictyol	−96.51	−74.79	−86.14	−87.82	−71.77
Isorhamnetin	−88.11	−67.44	−84.82	−81.17	−67.33
Isorhamnetin 3-*O*-rutinoside	−71.10	−61.11	−101.03	−36.47	−53.52
Kaempferol 3-*O*-glucoside	−78.10	−68.85	−91.81	−84.59	−60.37
Quercetin 3-*O*-glucoside	−64.92	−74.13	−101.60	−85.85	−68.56
Rutin	−33.14	−73.52	−106.18	−60.94	−63.66

**Fig. 2:**
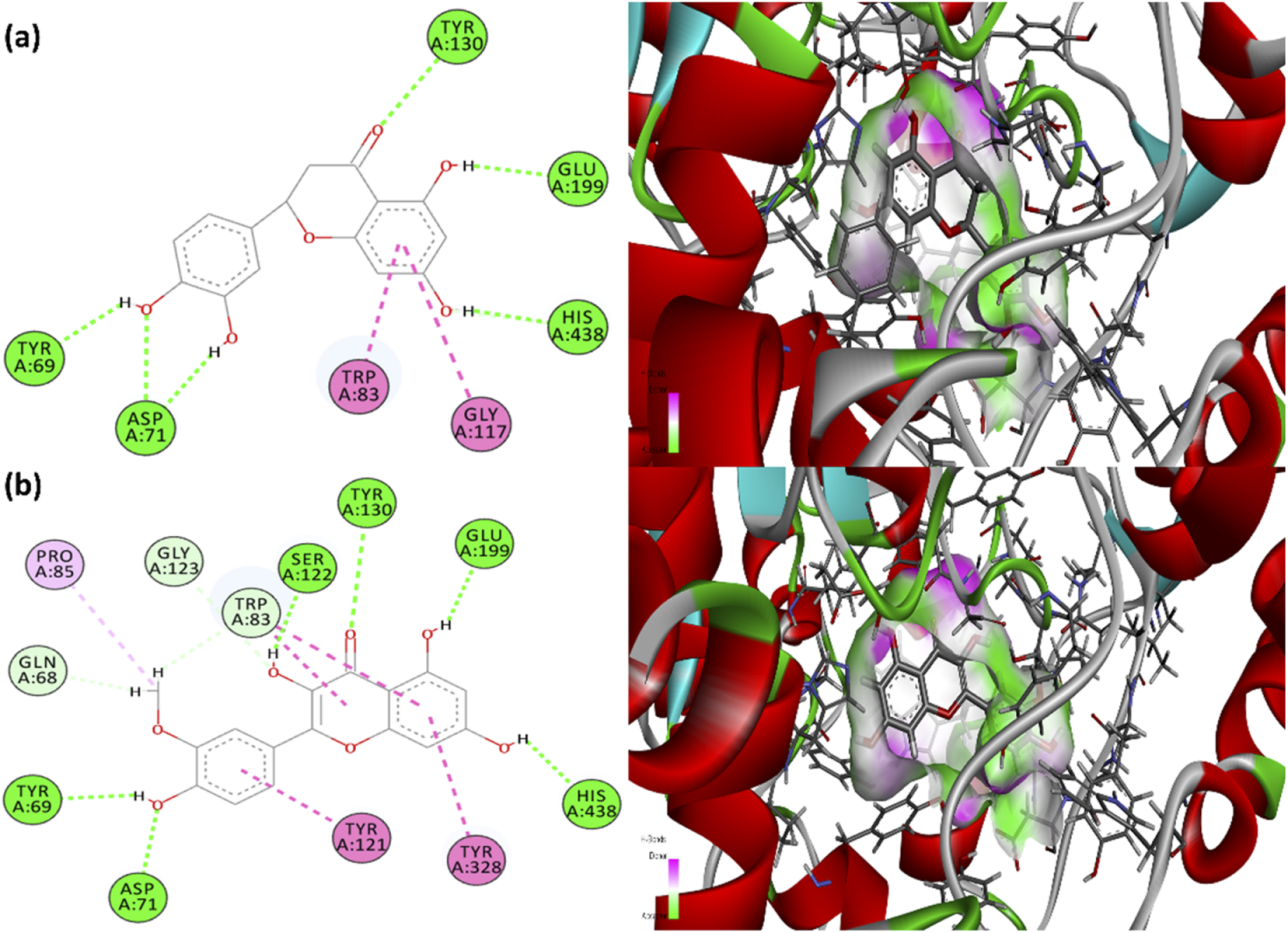
Two- and three-dimensional interaction representations show how the two highest-ranked compounds bind within the binding site of AChE. (a) Eriodictyol (b) isorhamnetin (light green: carbon–hydrogen bond, green: hydrogen bond, pink: pi–alkyl interaction, purple: pi–pi interaction).

Fig. 2 shows the two- and three-dimensional binding interactions between AChE and the two molecules with the best docking scores. Primary stabilization of the eriodictyol-AChE complex is established by a robust network of five hydrogen bonds (Fig. 2a). The carbonyl group of the chromenone ring forms a hydrogen bond acceptor interaction with Tyr130. Additionally, multiple hydroxyl groups in the chromenone structure form hydrogen bonds with several critical amino acid residues such as Tyr69, Asp71, Glu199, and His438. The docking profile of isorhamnetin with AChE reveals a complex binding orientation stabilized by a multifaceted network of noncovalent interactions (Fig. 2b). The stability of the complex is primarily driven by a robust series of hydrogen bonds, where the hydroxyl groups and the carbonyl oxygen of the flavonoid skeleton interact with critical residues including Tyr69, Asp71, Ser122, Tyr130, Glu199, and His438. These interactions are further reinforced by carbon–hydrogen bonds with Gln68, Trp83, and Gly123, which provide additional anchoring within the binding site. Furthermore, the aromatic rings of isorhamnetin facilitates essential hydrophobic contacts, characterized by pi–pi interactions with the aromatic side chains of Tyr121, Tyr328, and Trp83.

**Fig. 3:**
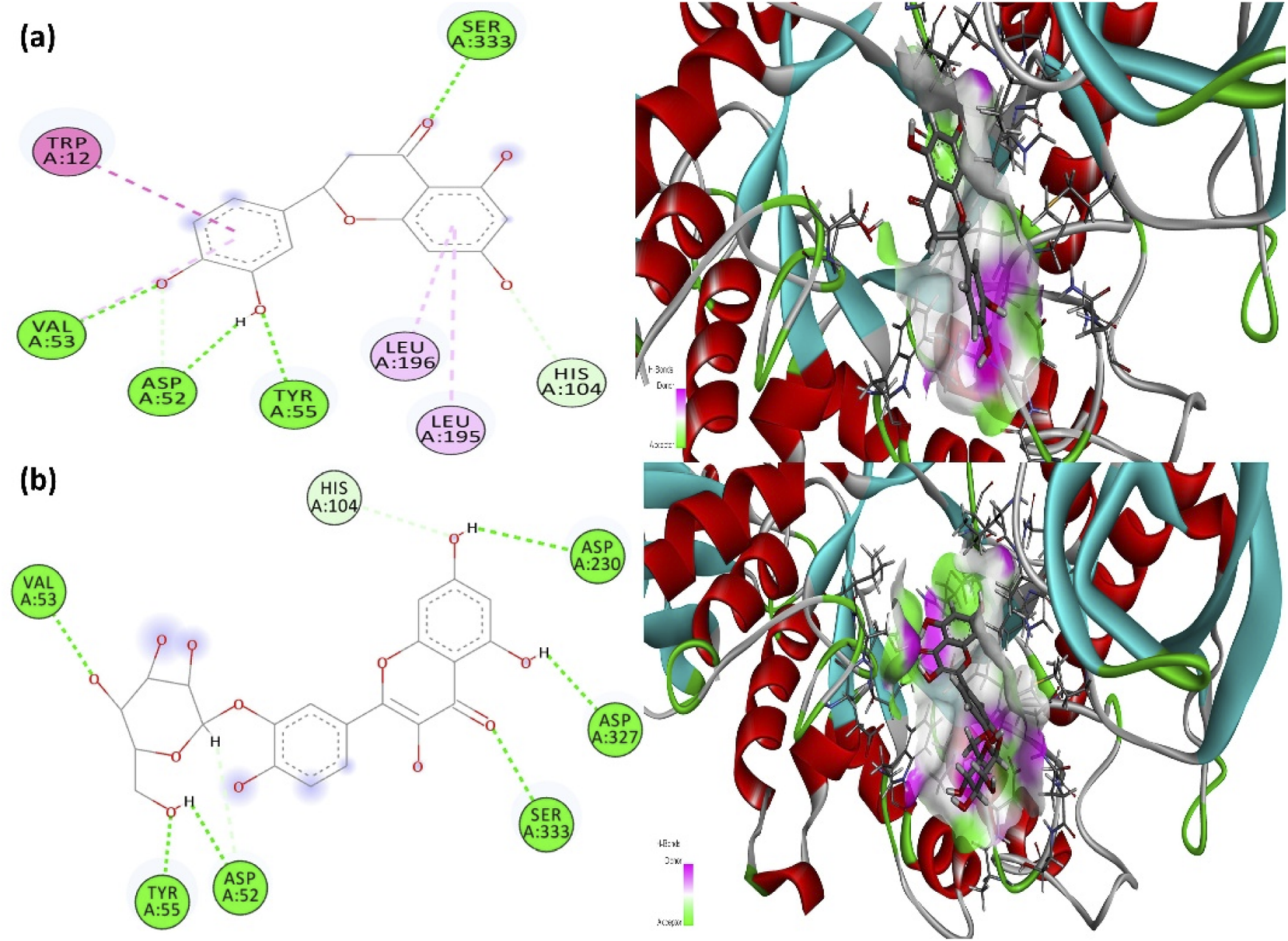
Two- and three-dimensional interaction representations show how the two highest-ranked compounds bind within the binding site of AML. (a) Eriodictyol (b) quercetin 3-*O*-glucoside (light green: carbon–hydrogen bond, green: hydrogen bond, pink: pi–alkyl interaction, purple: pi–pi interaction).

Molecular docking analysis of eriodictyol with AML shows that it is achieved *via* a series of hydrogen bonds in which the hydroxyl groups and carbonyl oxygen interact with residues Val53, Tyr55, and Ser333 (Fig. 3a). These polar interactions are further supplemented by carbon–hydrogen bonds involving Asp52 and His104, which provide additional orientation within the binding site. Furthermore, the aromatic structure of eriodictyol forms significant hydrophobic contacts, including a pi–pi interaction with Trp12 and pi–alkyl interactions with Leu195 and Leu196. The binding mode between quercetin 3-*O*-glucoside and AML indicates that the glucoside part of the molecule and the hydroxyl groups of the flavonoid core interact with various residues, particularly Asp52, Val53, Tyr55, Asp230, Asp327, and Ser333, and are stabilized by hydrogen bonding (Fig. 3b).

**Fig. 4:**
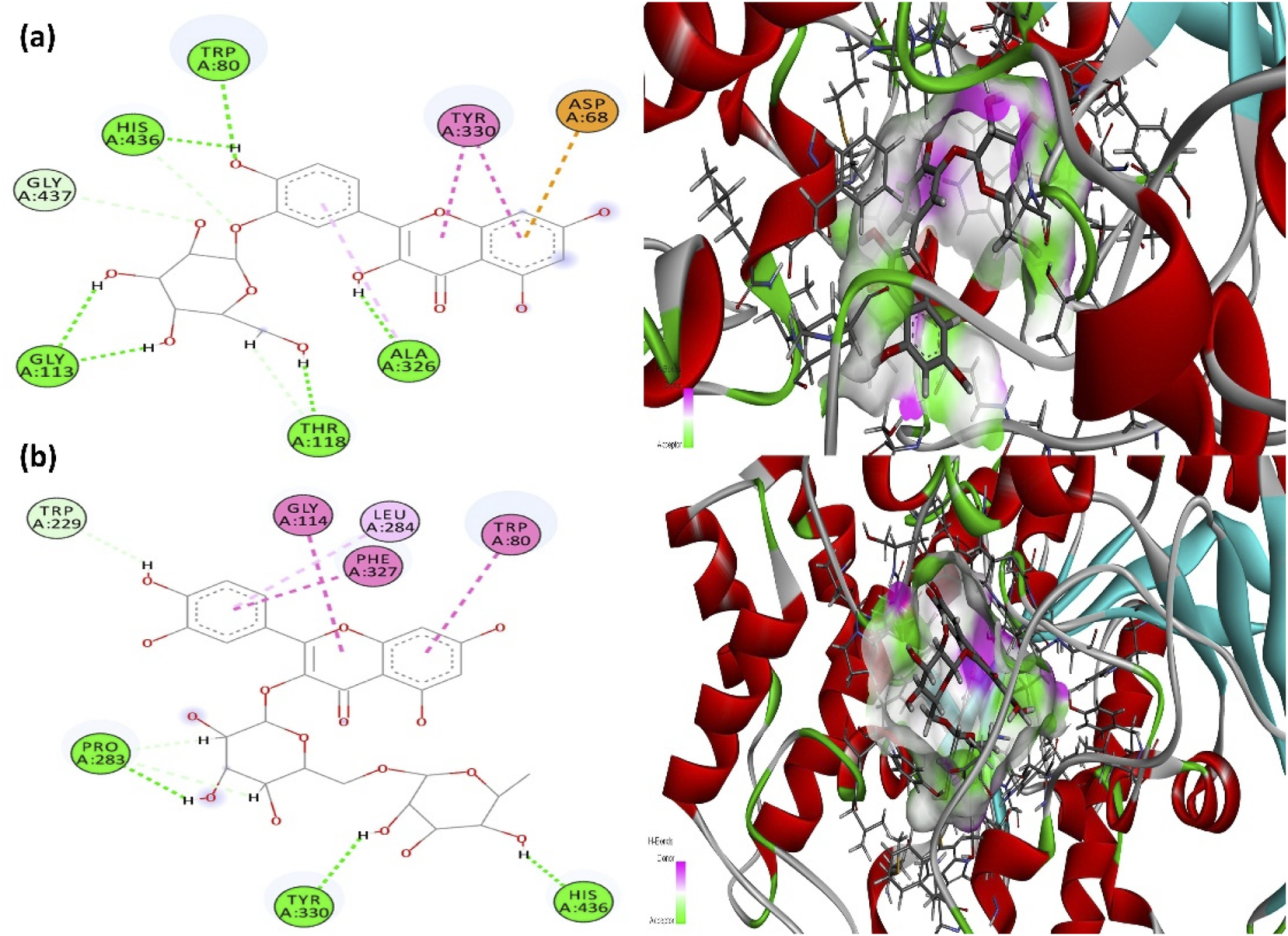
Two- and three-dimensional interaction representations show how the two highest-ranked compounds bind within the binding site of BChE (a) quercetin 3-*O*-glucoside (b) rutin (light green: carbon–hydrogen bond, green: hydrogen bond, pink: pi–alkyl interaction, purple: pi–pi interaction).

Docking results show that quercetin 3-*O*-glucoside forms multiple hydrogen bonds between the hydroxyl groups on the ligand within the BChE binding pocket, specifically between Trp80, Gly113, Thr118, Ala326, and His436 (Fig. 4a). These interactions are further enhanced by additional carbon–hydrogen interactions with Thr118, His436, and Gly437. In addition, the aromatic structure is stabilized through hydrophobic and ionic interactions, including pi–pi stacking with Tyr330, a pi–anion interaction with Asp68, and a pi–alkyl contact with Ala326. Rutin, on the other hand, established a hydrogen bond network with Pro283, Tyr330, and His436 in the binding pocket of BChE (Fig. 4b). These are further strengthened by carbon–hydrogen bonds with Pro283 and Trp229, providing significant attachment to the enzyme's binding pocket. Moreover, the aromatic ring of rutin participates in a few critical hydrophobic interactions, including pi–pi stacking with Trp80 and Phe327, amide–pi stacked interaction with Gly114, and pi–alkyl contacts with Leu284.

**Fig. 5:**
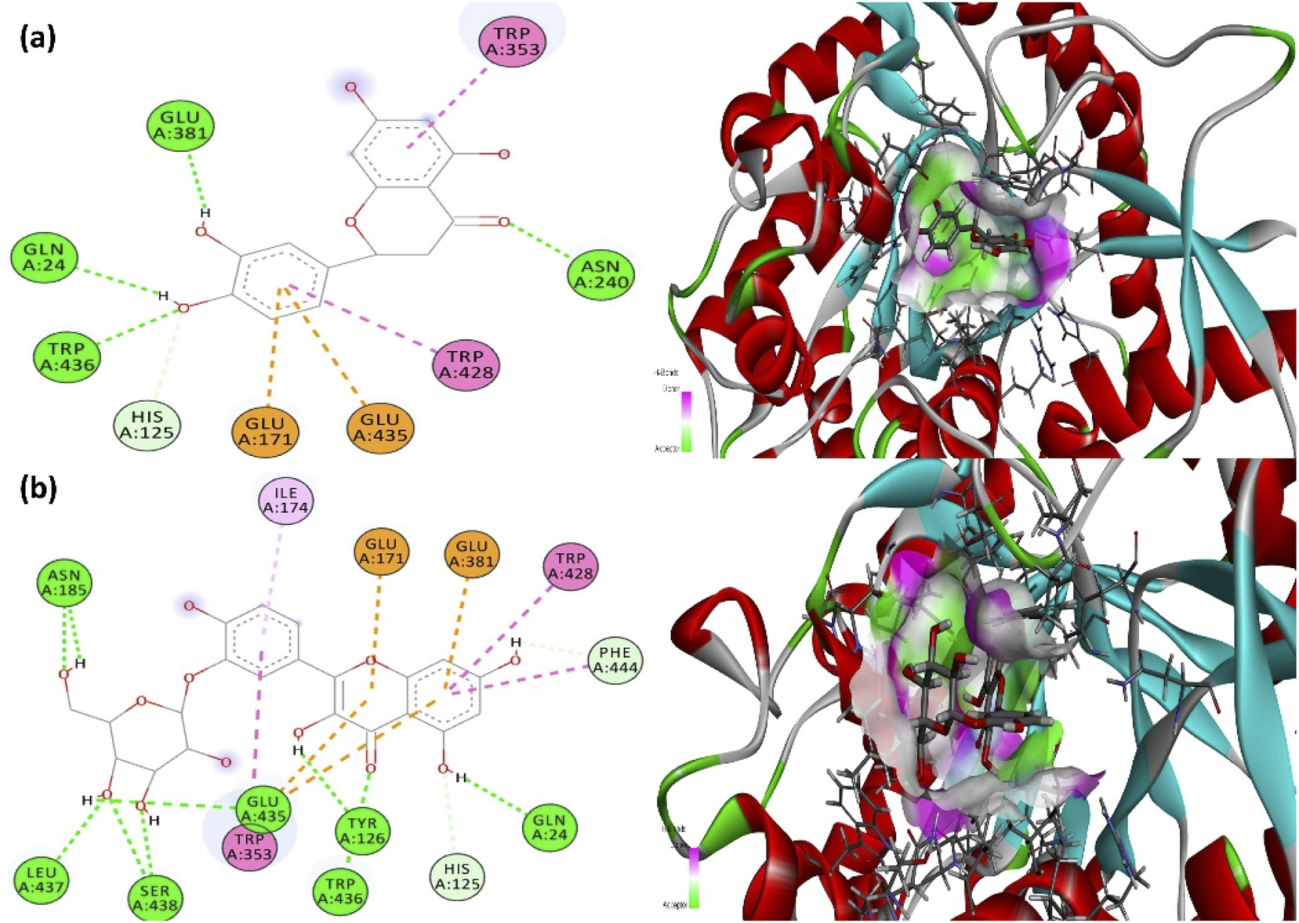
Two- and three-dimensional interaction representations show how the two highest-ranked compounds bind within the binding site of GLC. (a) Eriodictyol (b) quercetin 3-*O*-glucoside (light green: carbon–hydrogen bond, green: hydrogen bond, pink: pi–alkyl interaction, purple: pi–pi interaction, orange: pi–anion).

The binding mode of eriodictyol with GLC indicates that the ligand's ring structures interact with residues Gln24, Glu381, Trp436, and Asn240 (Fig. 5a). These polar interactions are completed by a carbon–hydrogen bond with His125, which further stabilizes the ligand within the binding pocket. Furthermore, the aromatic rings of eriodictol participates in important hydrophobic and ionic stabilization, including pi–pi interactions with Trp353 and Trp428, respectively, and pi–anion interactions with the carboxylate groups of Glu171 and Glu435. Quercetin 3-*O*-glucoside is stabilized by multiple hydrogen bonds with GLC (Gln24, Tyr126, Asn185, Glu435, Leu437 and Ser438) and further strengthened by carbon–hydrogen bonds with His125 and Trp436 (Fig. 5b). The aromatic rings of the ligand formed pi–anion contacts with Glu171, Glu381 and Glu435, and pi–pi interactions with Trp353 and Trp428.

**Fig. 6:**
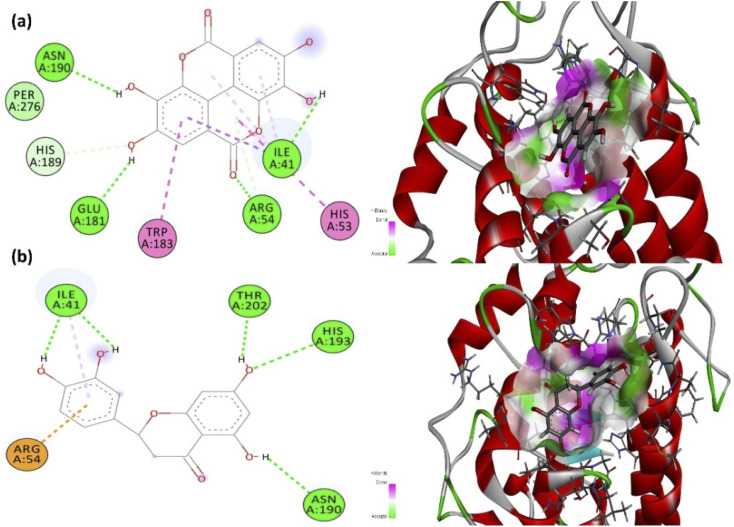
Two- and three-dimensional interaction representations show how the two highest-ranked compounds bind within the binding site of TYR. (a) Ellagic acid (b) eriodictyol (light green: carbon–hydrogen bond, green: hydrogen bond, pink: pi–alkyl interaction, purple: pi–pi interaction, orange: pi–cation).

The binding mode of ellagic acid in the TYR binding site indicates that the ligand interacts with the residues Ile41, Arg54, Glu181, and Asn190 (Fig. 6a). These interactions are further supported by carbon–hydrogen bonds and pi–donor hydrogen bonds involving His189 and Arg54, and van der Waals forces with Per276, which ensures the molecule's firm fixation. Furthermore, the aromatic rings of ellagic acid forms significant hydrophobic contacts, including pi–sigma interactions with Ile41, pi–pi interaction with His53 and Trp183, and pi–alkyl interactions with Ile41. Eriodictiol, on the other hand, forms four hydrogen bonds by directly interacting with the side chains of TYR, Ile41, Thr202, His193, and Asn190 (Fig. 6b).

### 3.8 MD simulations

**Fig. 7:**
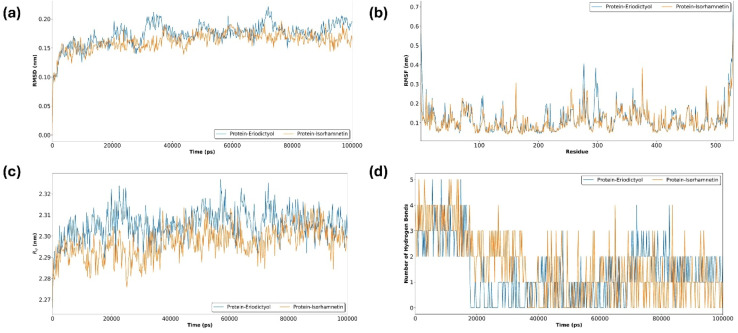
Molecular dynamics simulation analysis of AChE in complex with the two most potent molecules. (a) Root mean square deviation (RMSD), (b) root mean square fluctuation (RMSF), (c) radius of gyration, and (d) hydrogen bond analysis.

The conformational stability and complex binding properties of AChE with selected ligands were evaluated using MD simulations lasting 100 ns, and the binding trajectories of eriodictol and isorhamnetin to the enzyme are presented Fig. 7. As seen from the RMSD graph (Fig. 7a), both complexes attained structural equilibrium at around 20 ns; however, while the fluctuation of the isorhamnetin complex was lower and more constant (around 0.15 nm) than eriodictyol, the structural properties of the latter were more rigid, based on their *R*_g_ values (Fig. 7c), where the isorhamnetin complex had a more compact state (∼2.29 nm). In contrast, RMSF values for both complexes (Fig. 7b) were virtually unchanged throughout the simulation with variations of less than 0.3 nm. The hydrogen bonds (Fig. 7d) reveal the dominance of isorhamnetin in terms of its better stability and maintenance of 2–4 bonds compared to the transient decrease in bonding of eriodictyol during the middle stage of the simulation.

**Fig. 8:**
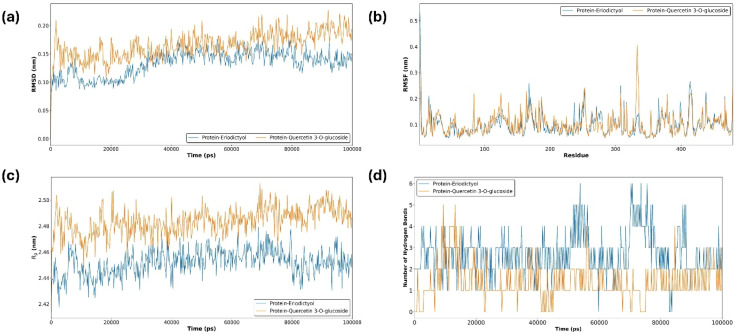
Molecular dynamics simulation analysis of AML in complex with the two most potent molecules. (a) Root mean square deviation (RMSD), (b) root mean square fluctuation (RMSF), (c) radius of gyration, and (d) hydrogen bond analysis.

RMSD obtained from MD simulation analysis for the complexes of eriodictol and quercetin 3-*O*-glucoside with AML indicates that eriodictyol forms a more stable complex due to its low deflection (∼0.14 nm), whereas quercetin 3-*O*-glucoside shows higher deflection (∼0.18 nm) (Fig. 8a). This trend is also validated by the *R*_g_ of the eriodictyol complex, which is smaller (∼2.45 nm) than that of the slightly larger quercetin 3-*O*-glucoside complex (∼2.49 nm) (Fig. 8c); the RMSF analysis demonstrates greater flexibility of the residues in the surrounding of 335 for the quercetin 3-*O*-glucoside complex (Fig. 8b). The results of hydrogen bonding analysis show that eriodictyol sustains stable bonding (2 to 5 bonds), while quercetin 3-*O*-glucoside exhibits transient hydrogen bonding (Fig. 8d).

**Fig. 9:**
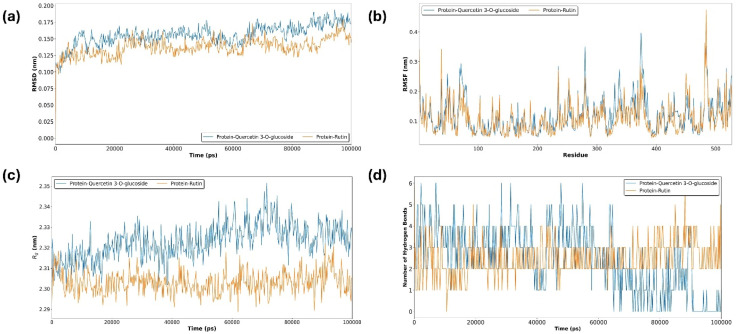
Molecular dynamics simulation analysis of BChE in complex with the two most potent molecules. (a) Root mean square deviation (RMSD), (b) root mean square fluctuation (RMSF), (c) radius of gyration, and (d) hydrogen bond analysis.

RMSD values of complexes of BChE reveal that both the complexes were at equilibrium, but the one with rutin was more stable with less deviation (∼0.14 nm), whereas quercetin 3-*O*-glucoside had a gradual rise to ∼0.18 nm (Fig. 9a). This is further supported by *R*_g_ analysis, where the rutin complex remained more compact (∼2.30 nm) (Fig. 9c), while quercetin 3-*O*-glucoside exhibited higher structural expansion and variability, and showed a slight increase in local flexibility near residues 80 and 380 in the RMSF profiles (Fig. 9b). Hydrogen bond analysis shows that rutin maintained a stable network of 1–4 interactions throughout the simulation, further highlighting its stronger binding persistence, whereas quercetin 3-*O*-glucoside lost a significant portion of its interactions after 60 ns (Fig. 9d).

**Fig. 10:**
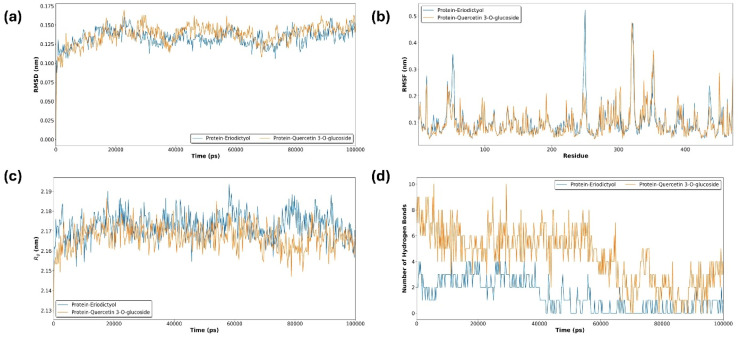
Molecular dynamics simulation analysis of GLC in complex with the two most potent molecules. (a) Root mean square deviation (RMSD), (b) root mean square fluctuation (RMSF), (c) radius of gyration, and (d) hydrogen bond analysis.

The RMSD results of the two complexes show that both eriodictyol and quercetin 3-*O*-glucoside achieve equilibrium after 20 ns of simulation using GLC, with the deviation being constant between 0.12 to 0.15 nm, thus no conformational distortion occurs (Fig. 10a). These results are supported by the *R*_g_ (Fig. 4c) and RMSF (Fig. 10b), showing a tightly packed protein molecule (around 2.17 nm) that exhibits restricted mobility, mainly in the periphery cycle's part. Hydrogen bonding analysis shows that quercetin 3-*O*-glucoside forms a tighter hydrogen bonding network and retains around 4 to 8 hydrogen bonds through the simulation time (Fig. 10d).

**Fig. 11:**
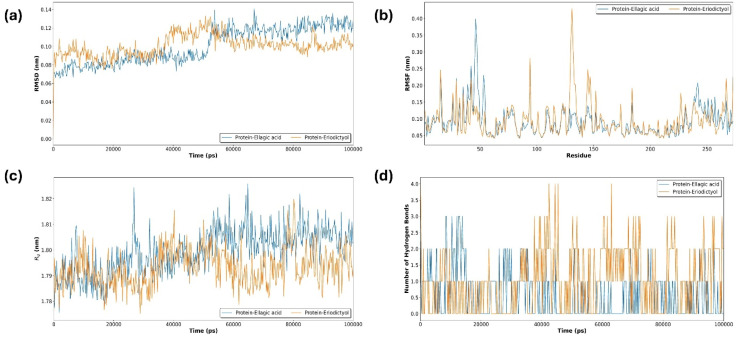
Molecular dynamics simulation analysis of TYR in complex with the two most potent molecules. (a) Root mean square deviation (RMSD), (b) root mean square fluctuation (RMSF), (c) radius of gyration, and (d) hydrogen bond analysis.

RMSD analysis of the TYR complex shows that both ellagic acid and eriodictyol complexes are relatively stable, with a deflection of less than 0.14 nm; however, eriodictyol exhibits increasing stability (∼0.10 nm) in the last 40 nanoseconds, while ellagic acid shows an increase in the middle of the trajectory (Fig. 11a). RMSF and *R*_g_ values confirm that the enzyme maintains a compact and rigid structure (1.78–1.82 nm) and exhibits only localized flexibility near residues 50 and 130 (Fig. 11b and c). Specifically, hydrogen bond analysis shows stronger binding persistence for eriodictyol, forming a more coherent network containing up to 4 hydrogen bonds, while ellagic acid exhibited fewer and more transient interactions.

The free energy terms of the interactions between the studied enzyme-ligand was quantitatively analyzed by means of the gmx_MMPBSA software, demonstrating specific thermodynamic characteristics responsible for their inhibitory action (Table 8). Among all studied interactions, rutin displayed the most pronounced affinity for BChE, yielding an exceptional *Δ*_TOTAL_ of −41.53 kcal mol^−1^. This high-potency binding is predominantly attributed to a superior van der Waals contribution (*Δ*_VDWAALS_ = −60.55 kcal mol^−1^), suggesting that rutin's specific molecular structure achieves optimal steric complementarity within the BChE binding pocket. In the case of AChE, isorhamnetin and eriodictyol exhibited robust stability with total binding energies of −26.64 kcal mol^−1^ and −24.26 kcal mol^−1^, respectively, where non-polar contributions remained the primary driving force for complexation. Importantly, the binding profile for GLC was uniquely characterized by strong electrostatic forces in the case of quercetin 3-*O*-glucoside (Δ*E*_EL_ = −57.23 kcal mol^−1^), resulting in a favorable total energy of −28.38 kcal mol^−1^, which significantly surpassed the −17.04 kcal mol^−1^ observed for eriodictyol.

**Table 8:** Energy contribution profiles of the investigated compounds in complex with the five target enzymes

Molecules/parameters	*Δ*_VDWAALS_ (kcal mol^−1^)	Δ*E*_EL_ (kcal mol^−1^)	Δ*E*_GB_ (kcal mol^−1^)	Δ*G*_GAS_ (kcal mol^−1^)	Δ*G*_SOLV_ (kcal mol^−1^)	*Δ*_TOTAL_ (kcal mol^−1^)
**ACHE (4EY5)**						
Eriodictyol	−30.04 ± 0.59	−19.94 ± 2.45	29.83 ± 0.08	−49.98 ± 2.52	25.73 ± 0.11	−24.26 ± 2.52
Isorhamnetin	−36.38 ± 0.98	−24.41 ± 6.93	39.26 ± 2.81	−60.79 ± 7.00	34.15 ± 2.81	−26.64 ± 7.54

**AML (9FZ3)**						
Eriodictyol	−13.48 ± 0.95	−28.83 ± 3.68	24.69 ± 2.64	−42.32 ± 3.80	21.86 ± 2.64	−20.45 ± 4.63
Quercetin 3-*O*-glucoside	−22.54 ± 0.87	−26.02 ± 6.90	36.37 ± 2.36	−48.56 ± 6.96	32.58 ± 2.36	−15.98 ± 7.35

**BCHE (4TPK)**						
Quercetin 3-*O*-glucoside	−37.35 ± 1.79	−33.55 ± 4.91	49.53 ± 1.32	−70.90 ± 5.22	44.01 ± 1.32	−26.89 ± 5.39
Rutin	−60.55 ± 1.48	−26.00 ± 5.42	53.40 ± 1.98	−86.54 ± 5.62	45.02 ± 1.98	−41.53 ± 5.96

**GLC (2RGL)**						
Eriodictyol	−19.40 ± 0.96	−21.39 ± 1.28	26.72 ± 3.90	−40.79 ± 1.59	23.75 ± 3.90	−17.04 ± 4.21
Quercetin 3-*O*-glucoside	−28.04 ± 1.40	−57.23 ± 4.10	62.53 ± 3.34	−85.27 ± 4.34	56.89 ± 3.34	−28.38 ± 5.47

**TYR (1WX2)**						
Ellagic acid	−16.07 ± 0.21	−6.96 ± 4.47	16.19 ± 0.82	−23.03 ± 4.48	14.44 ± 0.85	−8.59 ± 4.56
Eriodictyol	−17.91 ± 0.97	−15.39 ± 4.16	23.69 ± 2.22	−33.30 ± 4.27	20.88 ± 2.22	−12.42 ± 4.81

Conversely, eriodictyol demonstrated superior binding efficiency against AML and TYR, achieving *Δ*_TOTAL_ values of −20.45 kcal mol^−1^ and −12.42 kcal mol^−1^, respectively, outperforming its counterparts in these specific targets. A comprehensive analysis of the energetic components indicates that the favorable gas-phase energies (Δ*G*_GAS_), reaching values as low as −86.54 kcal mol^−1^ for rutin, act as the fundamental stabilizers that effectively counteract the polar desolvation penalties (Δ*G*_SOLV_) associated with the transition of ligands from the aqueous environment to the protein binding pockets.

### 3.9 DFT calculations

Fig. S3 visually presents HOMO, LUMO and Electrostatic Potential (ESP) maps of selected seven compounds while Table S2 gives calculated electronic structural parameters for these compounds. The relation between chemical hardness and reactivity was illuminated with Maximum Hardness Principle.^69^ This principle, formulated by Pearson, a scientist who introduced the concept of chemical hardness, states that chemical systems behave in a way that allows them to reach their maximum hardness. This essentially implies that hardness is a measure of stability. In the light of this information and the data presented in the related table, it can be said that ellagic acid and eriodictyol are relatively more stable compared to other molecules. Another electronic structure principle is the Minimum Electrophilicity Principle illuminated the relation between electrophilicity index and stability.^70^ According to this principle, it can be noted that electrophilicity index is minimized in stable states. This electronic structure principle also predicts that eriodictyol is more stable than others. In the section related to the Molecular Docking calculations, it was seen that eriodictyol interacts more powerful with the biological systems representing AchE, AML and GLC enzymes. This molecule also has the lowest electron affinity among all molecules, and its electronegativity is lower than that of other molecules. It has been noted that isorhamnetin 3-*O*-rutinoside, quercetin 3-*O*-glucoside, and rutin molecules, which have lower chemical hardness values, interact more strongly with the biological system representing the BChE enzyme. These molecules appear to have relatively lower ionization energies. Combined DFT and docking analyses have shown that ellagic acid and eriodictyol, the most rigid molecules, may be more effective in inhibiting the Tyr enzyme.

## 4 Conclusions

This study provides the first comprehensive investigation of *Rubus caesius* roots collected from the village of Germab in the Akhal Kopetdag foothills of Turkmenistan, demonstrating that both aqueous (infusion) and ethanolic extracts are polyphenol-rich matrices with considerable biological potential. UHPLC-QqQ-MS/MS profiling confirmed the presence of characteristic phenolic constituents, particularly ellagic acid, flavonoid glycosides, and hydroxycinnamic acid derivatives, which have previously been reported to possess antioxidant and multifunctional bioactivities. The comparative extraction strategy indicated that solvent polarity influences both the qualitative and quantitative phytochemical composition of the extracts, and these compositional differences were reflected in the observed biological activity profiles. Specifically, the aqueous extract was characterized by higher levels of tannin-derived polar phenolics and exhibited superior antioxidant and metal-chelating activities, whereas the ethanolic extract showed enhanced flavonoid recovery and stronger enzyme-inhibitory and selective antimicrobial effects.

Significantly, the ethanolic extract appears particularly suitable for applications that require enrichment of flavonoid-related bioactive compounds, while the infusion extract reflects traditional preparation methods and may yield greater recovery of highly polar antioxidant constituents. This complementary, solvent-dependent behavior suggests that both preparations have practical value and may be selected based on the targeted pharmacological or functional application.

Across the *in vitro* assays performed, *R. caesius* root extracts exhibited strong antioxidant capacity, along with multifunctional enzyme-inhibitory effects against cholinesterases, carbohydrate-hydrolyzing enzymes, and tyrosinase. The observed activities were strongly associated with the phytochemical profile of the extracts, especially the abundance of ellagic acid, rutin, quercetin derivatives, and isorhamnetin glycosides, which have previously been associated with radical scavenging, metal chelation, and enzyme inhibition, although the contribution of individual compounds could not be established in the present study. In addition, measurable antibacterial and antifungal activities were observed, highlighting the potential of these extracts as natural antimicrobial agents or complementary ingredients in health-oriented formulations. Although the antimicrobial potency remained lower than standard synthetic drugs, the broad-spectrum inhibitory effects observed against several bacterial and filamentous fungal strains suggest that the roots contain phenolic metabolites that may contribute to the observed antimicrobial activity.

In summary, the present findings indicate that *R. caesius* roots from Turkmenistan represent a promising yet previously underexplored natural source of multifunctional phytochemicals with antioxidant, enzyme inhibitory, antibacterial, and antifungal potential. The integration of phytochemical profiling with multiple biological activity assays provides a strong scientific basis for future studies focused on a larger panel of extracts (including non-polar solvents), compound isolation, mechanistic evaluation, and the development of phytopharmaceutical, nutraceutical, cosmetic, or natural preservative applications derived from this species.

## Author contributions

Serdar Korpayev: investigation, methodology, formal analysis, writing – original draft, writing – review and editing. Emre Can Buluz: investigation, methodology, visualization, formal analysis. Savaş Kaya: investigation, methodology, visualization, formal analysis. Jasmina Glamočlija: investigation, methodology, writing – original draft, writing – review and editing. Neda Popović: investigation, methodology, writing – original draft, writing – review and editing. Uroš Gašić: investigation, methodology, writing – original draft, writing – review and editing. Dejan Stojković: investigation, methodology, writing – original draft, writing – review and editing. Hemra Hamrayev: investigation, methodology, resources. Mirap Agamuradov: investigation, methodology, resources. Sakina Yagi: investigation, methodology, formal analysis, writing – original draft, writing – review and editing. Gökhan Zengin: investigation, methodology, formal analysis, writing – original draft, writing – review and editing.

## Conflicts of interest

There are no conflicts to declare.

## Supplementary Material

RA-OLF-D6RA04860A-s001

## Data Availability

Data may be requested from the authors. Supplementary information (SI): Table S1. Analytical and validation parameters. Table S2. CDFT-derived electronic properties of selected seven compounds. Fig. S1. Calibration curves of gallic acid (for total phenolic content) and rutin (for total flavonoid content). Fig. S2. HOMO, LUMO and electrostatic potential (ESP) maps of selected seven compounds. Fig. S3. HOMO, LUMO and electrostatic potential (ESP) maps of selected seven compounds. See DOI: https://doi.org/10.1039/d6ra04860a.
