# Supplementary material for: Phenolic-rich Rubus caesius roots from Turkmenistan: in vitro antioxidant, enzyme inhibition, antibacterial, and antifungal properties and in silico analysis
Source: RSC Adv. 2026 Aug 11;16(40):46715–33. doi: 10.1039/d6ra04860a (PMC13459054; doi:10.1039/d6ra04860a)
Supplement: Supplementary file 1 [file RA-016-D6RA04860A-s001.pdf]

Supplementary Information (SI) for RSC Advances.
This journal is © The Royal Society of Chemistry 2026

Table S1. Analytical and validation parameters

**Compounds**	**Retention time, min**	**Precursor ions, *m/z***	**Product ions (*m/z*) with specied collision energies (eV)**	**Calibration range, mg/L**	**Equation,Y = A+B*X**	**R^2**	**LOD, mg/L**	**LOQ, mg/L**
**3-O-Caffeoylquinic acid**	5.00	353.103	191.28 (25)	0.01-1.00	Y = -1729+513670*X	0.9968	0.07	0.24
**5-O-Caffeoylquinic acid**	5.67	353.101	191.18 (25)	0.01-1.00	Y = -178+770960*X	0.9959	0.08	0.27
**Caffeic acid**	6.18	179.004	135.06 (18); 107.13 (18)	0.05-1.00	Y = 2169830*X	0.9950	0.08	0.28
**Rutin**	6.71	609.197	301.20 (32); 299.98 (42)	0.01-1.00	Y = -8085+4756710*X	0.9994	0.03	0.10
**Vitexin**	6.80	430.999	341.10 (25); 311.06 (27)	0.01-1.00	Y = -16625+8356270*X	0.9978	0.07	0.24
**p-Coumaric acid**	6.96	163.072	119.34 (20)	0.25-1.00	Y = 63215+94534*X	0.9976	0.06	0.19
**Quercetin 3-O-glucoside**	6.97	463.002	301.04 (23); 300.02 (29)	0.01-0.75	Y = -20398+4970500*X	0.9996	0.02	0.07
**Ellagic acid**	7.06	300.980	284.00(32); 300.04(30)	0.25-1.00	Y = 133641*X	0.9938	0.11	0.37
**Isorhamnetin 3-O-rutinoside**	7.13	622.827	313.95 (7); 299.96 (51)	0.01-1.00	Y = -1172+1290340*X	0.9996	0.02	0.08
**Kaempferol 3-O-glucoside**	7.41	447.008	284.03 (29); 255.03 (43)	0.01-1.00	Y = 9791+3433970*X	0.9978	0.06	0.20
**Eriodictyol**	8.62	286.974	150.93 (19); 135.02 (22)	0.01-0.75	Y = -25400+2419190*X	0.9963	0.06	0.20
**Naringenin**	9.39	271.036	151.01 (20); 107.07 (26)	0.01-1.00	Y = -29883+2692320*X	0.9993	0.03	0.11
**Hispidulin**	9.57	299.056	284.11 (35)	0.01-1.00	Y = 21735+14207700*X	0.9979	0.06	0.19
**Isorhamnetin**	9.73	314.989	299.95 (25)	0.01-0.75	Y = -43722+1639670*X	0.9966	0.06	0.19

Table S2. CDFT-derived electronic properties of selected seven compounds.

Molecules/ Parameters	E_{HOMO}	E_{LUMO}	Ionization Potential	Electron Affinity	Energy Gap (ΔE)	Electronegativity (χ)	Chemical Potential (μ)	Chemical Hardness (η)	Global Softness (σ)	Electrophilicity Index (ω)
**Ellagic acid**	-6.028	-1.723	6.028	1.723	4.305	3.876	-3.876	2.153	0.465	3.489
**Eriodictyol**	-5.456	-0.969	5.456	0.969	4.487	3.213	-3.213	2.244	0.446	2.300
**Isorhamnetin**	-5.289	-1.811	5.289	1.811	3.478	3.550	-3.550	1.739	0.575	3.624
**Isorhamnetin 3-O-rutinoside**	-4.841	-1.077	4.841	1.077	3.764	2.959	-2.959	1.882	0.531	2.326
**Kaempferol 3-O-glucoside**	-5.763	-1.871	5.763	1.871	3.892	3.817	-3.817	1.946	0.514	3.743
**Quercetin 3-O-glucoside**	-5.215	-1.510	5.215	1.510	3.705	3.363	-3.363	1.853	0.540	3.052
**Rutin**	-5.124	-1.346	5.124	1.346	3.778	3.235	-3.235	1.889	0.529	2.770

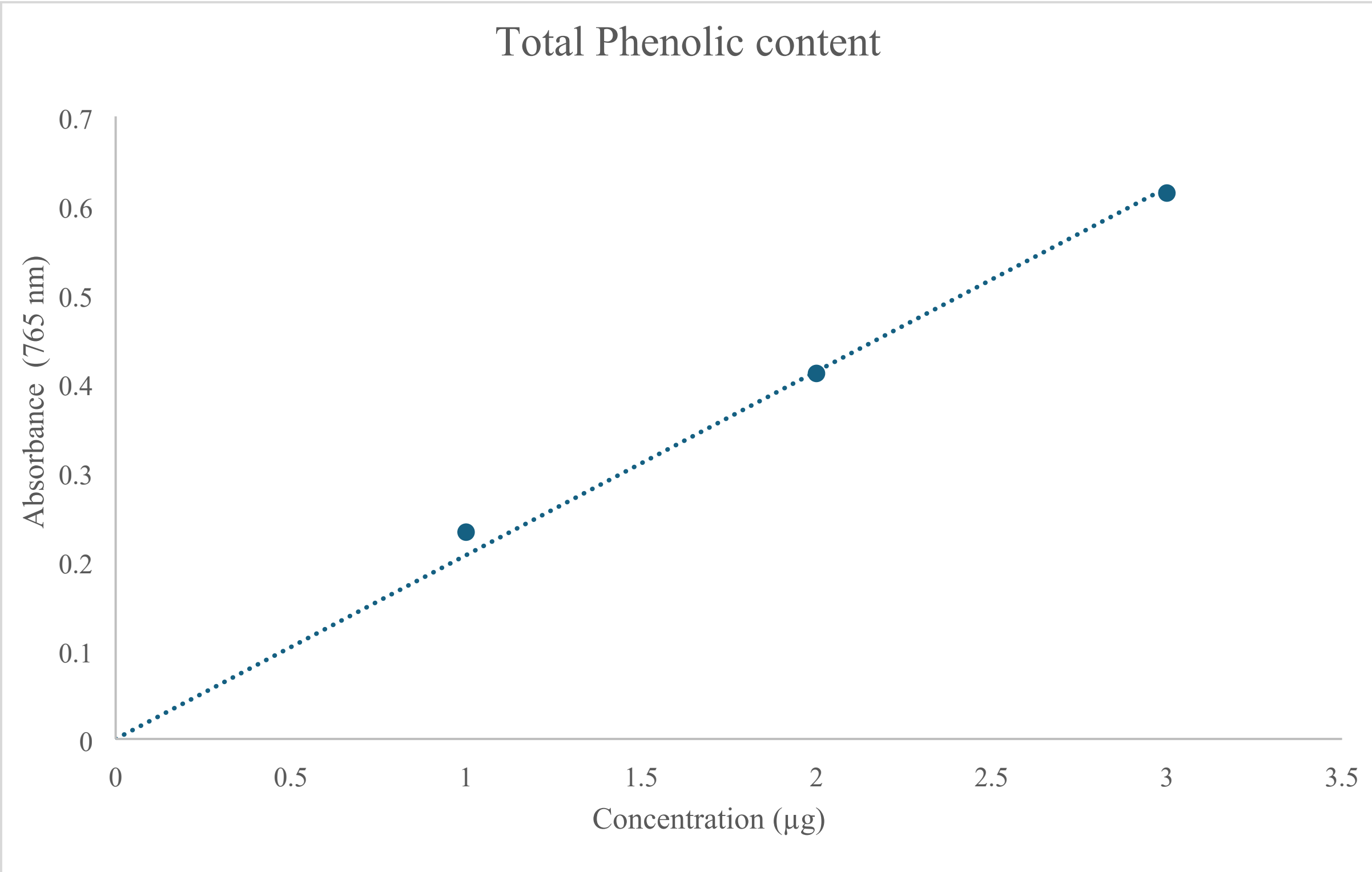

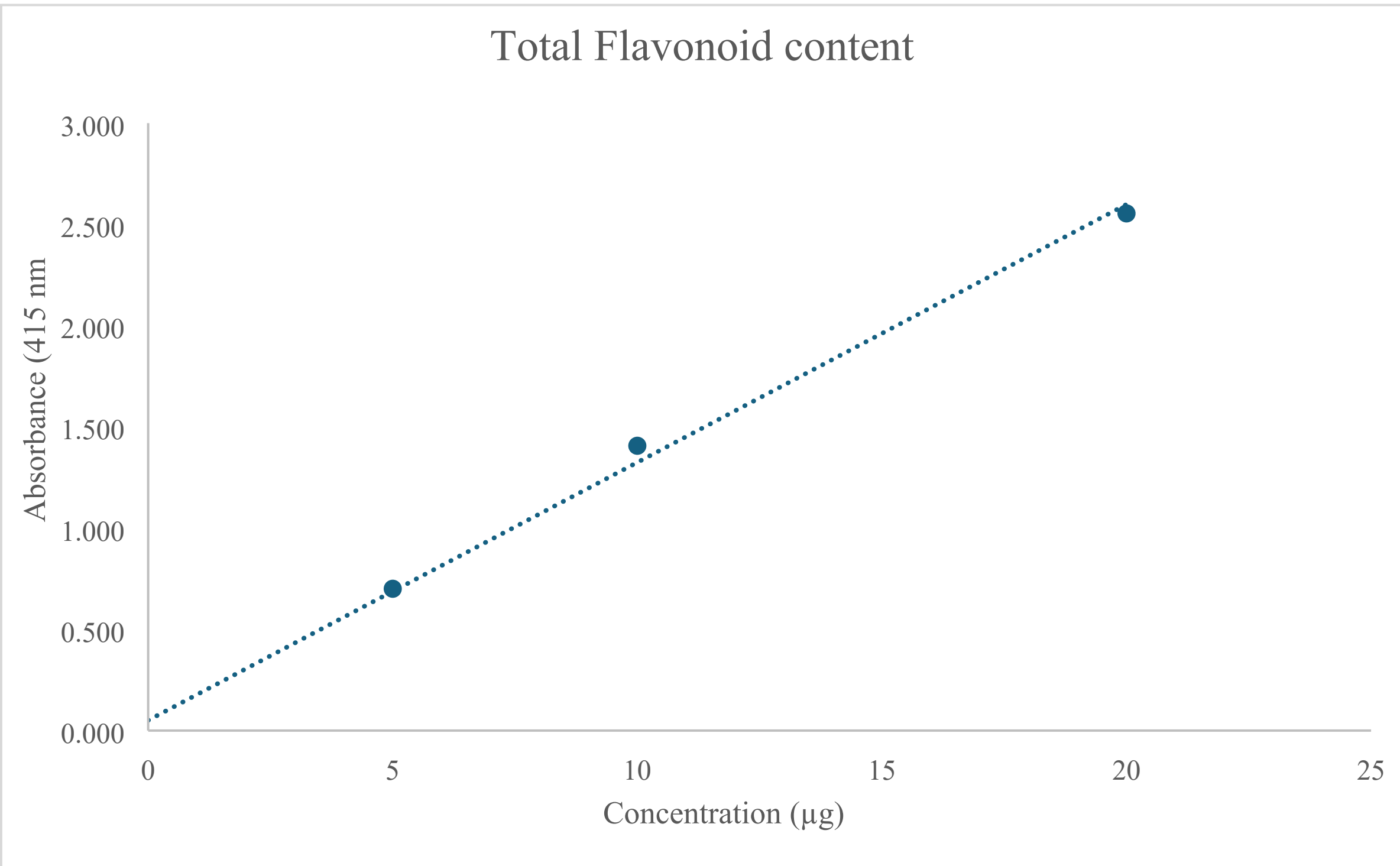

Figure S1. Calibration curves of gallic acid (for total phenolic content) and rutin (for total flavonoid content)

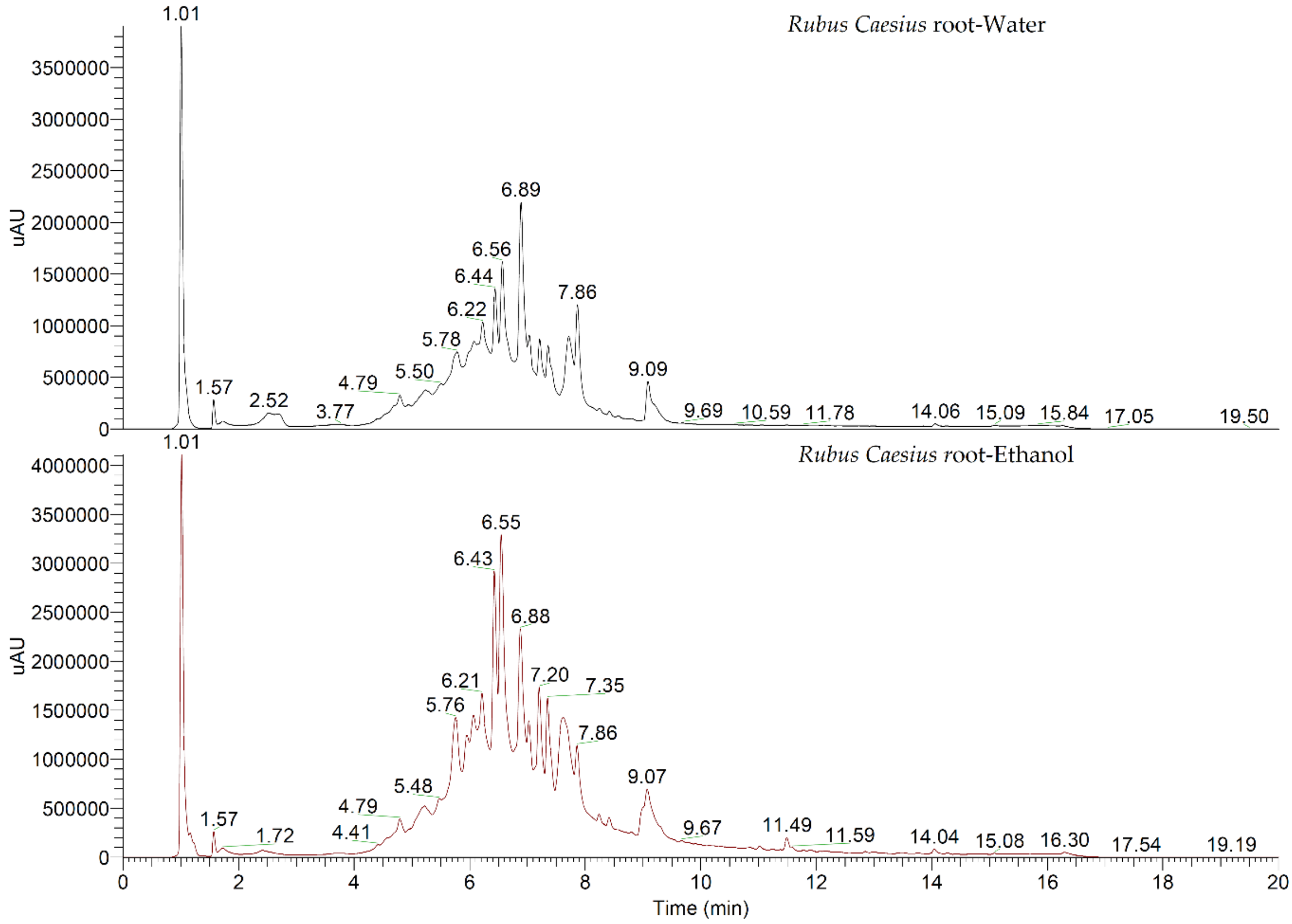

Figure S2. HOMO, LUMO and Electrostatic Potential (ESP) maps of selected seven compounds.

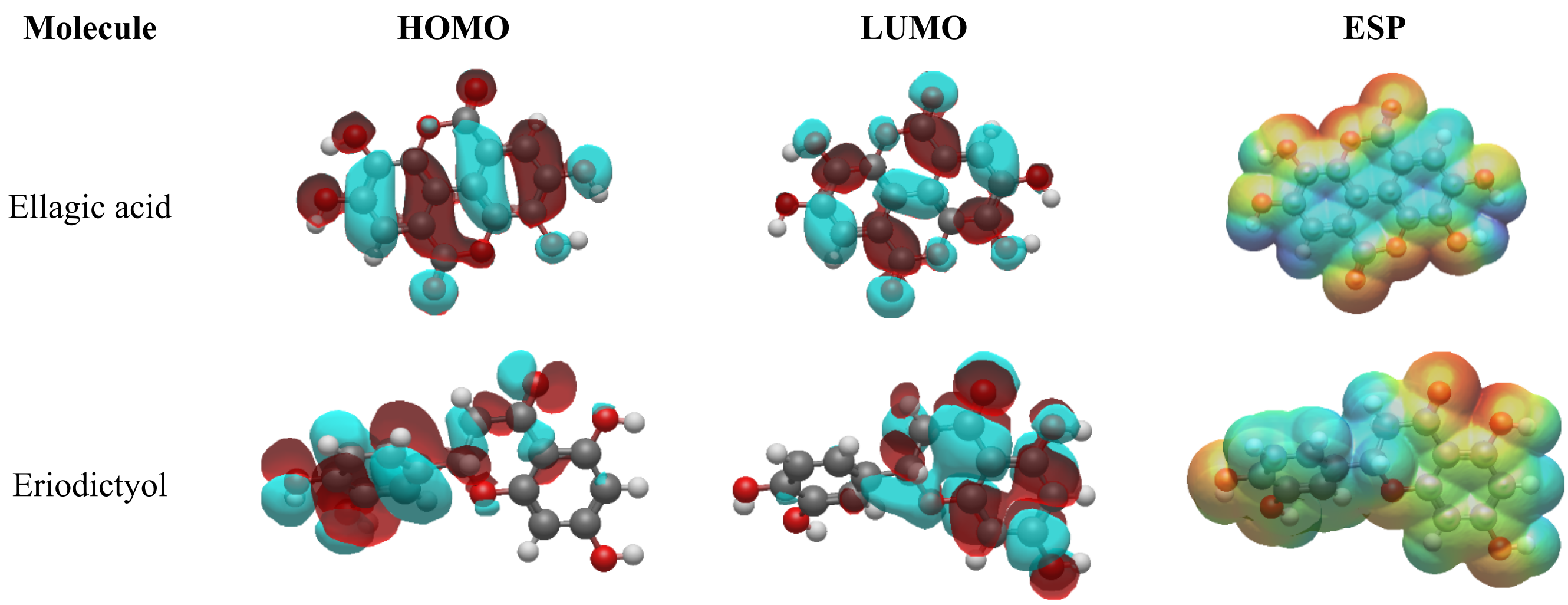

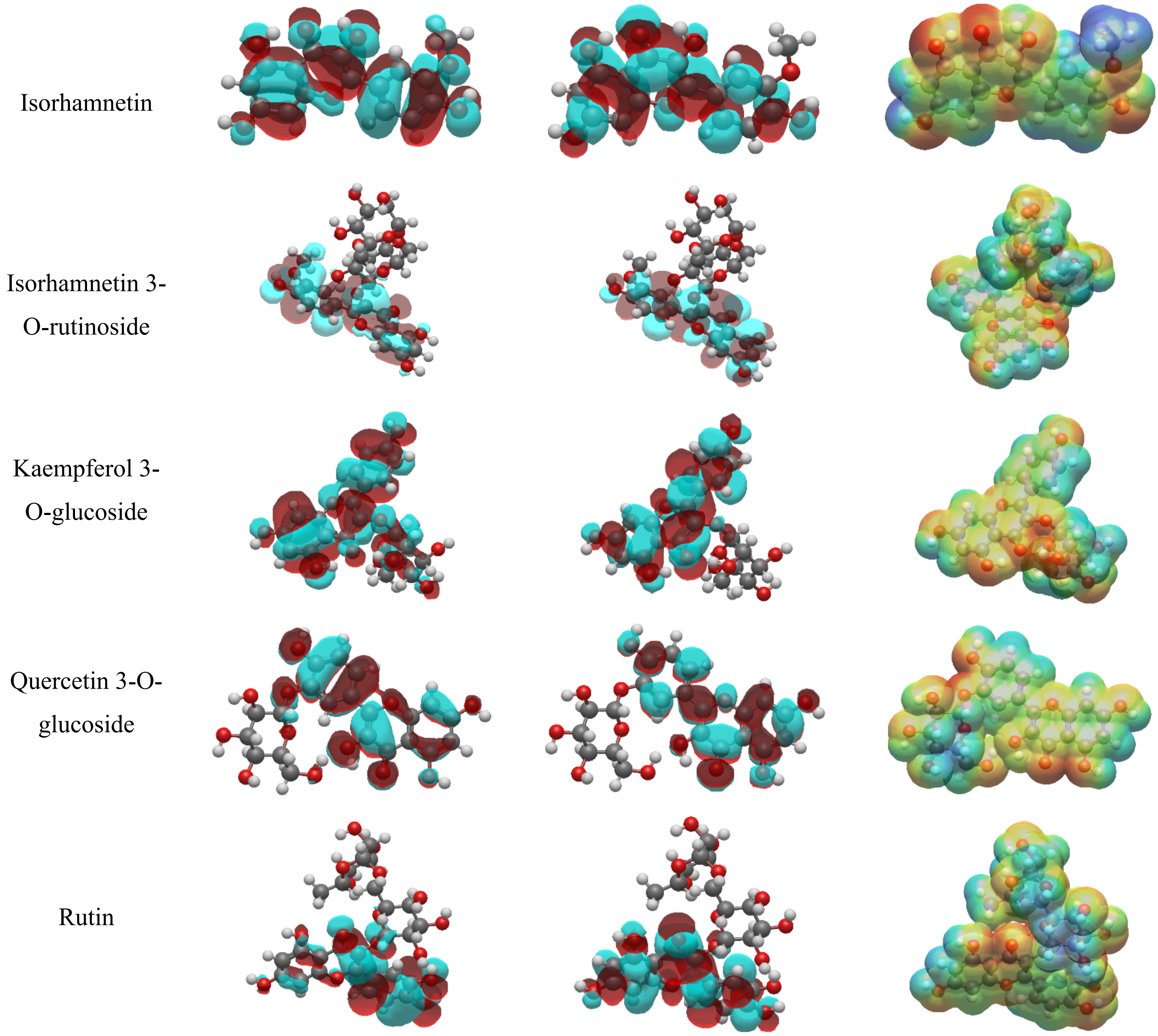

Figure S3. HOMO, LUMO and Electrostatic Potential (ESP) maps of selected seven compounds.
